# Genomic prediction for hastening and improving efficiency of forward selection in conifer polycross mating designs: an example from white spruce

**DOI:** 10.1038/s41437-019-0290-3

**Published:** 2020-01-22

**Authors:** Patrick R. N. Lenz, Simon Nadeau, Aïda Azaiez, Sébastien Gérardi, Marie Deslauriers, Martin Perron, Nathalie Isabel, Jean Beaulieu, Jean Bousquet

**Affiliations:** 1 Natural Resources Canada, Canadian Wood Fibre Centre, 1055 rue Du PEPS, P.O. Box 10380, QC, G1V 4C7 Canada; 2 Canada Research Chair in Forest Genomics, Institute of Systems and Integrative Biology, and Centre for Forest Research, Université Laval, 1030 Avenue de la Médecine, QC, G1V 0A6 Canada; 3 Ministère des Forêts, de la Faune et des Parcs, Gouvernement du Québec, Direction de la recherche forestière, 2700 rue Einstein, QC, G1P 3W8 Canada; 4 Natural Resources Canada, Laurentian Forestry Centre, 1055 rue Du PEPS, P.O. Box 10380, QC, G1V 4C7 Canada

**Keywords:** Plant breeding, Quantitative trait

## Abstract

Genomic selection (GS) has a large potential for improving the prediction accuracy of breeding values and significantly reducing the length of breeding cycles. In this context, the choice of mating designs becomes critical to improve the efficiency of breeding operations and to obtain the largest genetic gains per time unit. Polycross mating designs have been traditionally used in tree and plant breeding to perform backward selection of the female parents. The possibility to use genetic markers for paternity identification and for building genomic prediction models should allow for a broader use of polycross tests in forward selection schemes. We compared the accuracies of genomic predictions of offspring’s breeding values from a polycross and a full-sib (partial diallel) mating design with similar genetic background in white spruce (*Picea glauca*). Trees were phenotyped for growth and wood quality traits, and genotyped for 4092 SNPs representing as many gene loci distributed across the 12 spruce chromosomes. For the polycross progeny test, heritability estimates were smaller, but more precise using the genomic BLUP (GBLUP) model as compared with pedigree-based models accounting for the maternal pedigree or for the reconstructed full pedigree. Cross-validations showed that GBLUP predictions were 22–52% more accurate than predictions based on the maternal pedigree, and 5–7% more accurate than predictions using the reconstructed full pedigree. The accuracies of GBLUP predictions were high and in the same range for most traits between the polycross (0.61–0.70) and full-sib progeny tests (0.61–0.74). However, higher genetic gains per time unit were expected from the polycross mating design given the shorter time needed to conduct crosses. Considering the operational advantages of the polycross design in terms of easier handling of crosses and lower associated costs for test establishment, we believe that this mating scheme offers great opportunities for the development and operational application of forward GS.

## Introduction

Genomic selection (GS), also called genomic prediction, relies on genome-wide dense marker maps to model the entire complement of QTL effects across the genome (Meuwissen et al. 2001), but also relies on relatedness among the individuals making up the training and the target populations (Zhong et al. 2009; Zapata-Valenzuela et al. 2012; Lenz et al. 2017). The estimation of an individual genetic merit through its genomic-estimated breeding values (GEBVs) has been demonstrated to be a valuable tool for a wide variety of traits in several forest trees such as *Eucalyptus* (Resende et al. 2012b), pines (Resende et al. 2012a; Zapata-Valenzuela et al. 2012; Isik et al. 2016), and spruces (Beaulieu et al. 2014; Chen et al. 2018; Lenz et al. 2020). With GS, instead of relying only on registered pedigree that are often prone to errors (Doerksen and Herbinger 2008; Munoz et al. 2014; Godbout et al. 2017), tree breeders can use genetic markers information to build additive and non-additive genomic relationship matrices that more accurately reflect the true relationships between individuals and simultaneously account for contemporary as well as historical pedigree (Grattapaglia et al. 2018). Thus, more accurate estimates of genetic values can be obtained with GS, based on both additive and non-additive variances, making possible to potentially increase genetic gains. Now that a number of proof-of concept studies highlighted the large potential of GS for hastened and accurate breeding of forest trees, questions remain about how to most effectively deploy GS strategies, for example, which experimental or breeding design is best to use in combination with GS or should conventional breeding strategies be revisited in the light of GS?

Breeding of cross-pollinated tree species, such as conifers, has traditionally followed recurrent selection schemes (Burdon and Shelbourne 1971; White et al. 2007), with cumulative genetic improvement captured over successive generations. To produce large amounts of genetically improved seed, tree breeders have generally established open-pollinated seed orchards using genotypes selected in progeny tests. Several types of mating design can be used to produce progenies from which additive and non-additive genetic variances, heritability, and breeding values can be estimated (Falconer and Mackay 1996). Among the mating designs available, the polycross, originally introduced by Frandsen (1940) and by Tysdal et al. (1942), is considered as one of the most cost-effective (Kumar et al. 2007). In the polycross mating design, each female tree is pollinated with a common mix of pollen from a number of male trees. This design is easy to implement and provides reliable estimates of breeding values of the female parents, with opportunities for improving genetic gain due to the large number of realized parental combinations (Lambeth et al. 2001). It is generally assumed that the pollen of each of the males in the mix is equally effective in the pollination­ fertilization process (Fowler 1987), and progenies are considered as half-sibs if mating occurs randomly. However, departure from this assumption is possible, even when the pollen amount of each male parent is adjusted to account for differences in pollen viability. When this happens, an overestimation of both additive genetic variance and prediction of genetic gain can occur (e.g. El-Kassaby et al. 2011; Vidal et al. 2015). Reproductive success through pollen viability has been tested for many conifer species, thanks to the availability of DNA markers and pedigree reconstruction techniques (e.g. *Picea abies*, Schoen and Cheliak 1987; *Pseudotsuga mensiesii*, El-Kassaby and Ritland 1992; *Pinus radiata*, Kumar et al. 2007). The use of reconstructed pedigree from marker data or the realized genomic relationship matrix in GS modelling should account for differential parentage and reproductive success and hence, prevent underestimation of relatedness and the overestimation of genetic variances. Moreover, in polycross progeny tests, non-additive genetic variance could not be estimated from the maternal pedigree when the male parents are unknown. Again, pedigree reconstruction or GS modelling may allow estimating relatedness more accurately and better separating additive and non-additive variances (Gamal El-Dien et al. 2016).

Forward selection in progeny tests is a frequently used strategy in forest tree breeding programs. Thereby, selected offspring are directly used for crosses and establishment of the next breeding cycle or for deployment, while in backward selection, parents are selected based on the genetic merit of their offspring. The polycross mating design has conventionally been used for backward selection of the female parents, but more recently the forward selection of their offspring has gained interest. Ruotsalainen and Lindgren (1998) showed that forward selection resulted in higher genetic gains than backward selection in polycross progeny tests, especially for traits harbouring high heritabilities and for larger polycross families. Moreover, from results of stochastic simulations, Burdon and Kumar (2004) concluded that provided the number of forward selections from the polycross progeny test to establish seed orchards is ≥15, any expected advantage of forward selection will almost always be realized for traits of moderate heritability (*h*^2^ ≥ 0.4), despite relatively imprecise estimation of offspring’s breeding values when the male parents are unknown. The use of genetic markers to recover paternity identity as a means of improving the accuracy of forward selections’ breeding values and to better manage inbreeding was first proposed by Lambeth et al. (2001) for tree breeding applications. Following paternity recovery in a maritime pine polycross progeny test, Vidal et al. (2017) found slightly higher genetic gains for forward selection than for backward selection with equal constraints on genetic diversity. We propose that the additional level of information provided by the genomic relationship matrix and the potentially enhanced accuracy of offspring’s genetic values obtained with GS models may further improve genetic gains in forward selection.

White spruce (*Picea glauca* [Moench] Voss) is one of the most commercially important conifer species and one of the most planted forest trees in Canada. Breeding programs have been active in most of the Canadian provinces since the 1960s (Mullin et al. 2011). Breeding strategies have evolved over generations with most recent use of partial diallel mating designs (i.e. biparental crosses resulting in full-sib progeny) and vegetative reproduction of genetically improved material using somatic embryogenesis and cuttings (Park et al. 2016). Full-sib progeny tests have thus been established (Beaulieu 1996), which makes it possible to estimate not only additive but also non-additive genetic variances, narrow-sense and broad-sense heritabilities as well as breeding and genetic values of progeny trees for adaptive and commercially important traits. Extensive genomic resources have also been developed for white spruce over the last decade (Rigault et al. 2011; Pavy et al. 2013a, 2013b, 2017). The availability of large numbers of high quality SNPs covering much of the white spruce transcriptome as well as large phenotypic databases from white spruce polycross and full-sib progeny tests from the same parental breeding population make it possible to build GS models from genomic profiles and compare the prediction accuracy obtained with these different mating designs for several quantitative traits.

The objectives of the present study were (1) to recover the paternity identity of white spruce polycross progeny and estimate the variation in the number of offspring for each pollen donor, (2) evaluate the bias in the additive genetic variance estimates when no differential male reproductive success is assumed in the polycross progeny test, that is, using the known maternal pedigree as compared with using the reconstructed full pedigree or the genomic relationship matrix, (3) build GS models for growth and wood quality traits using progeny tests from two different mating designs, that is polycross and full-sib (partial diallel) mating designs, to estimate offspring’s breeding values in the context of forward selection, (4) compare the prediction accuracy of conventional pedigree-based models versus GS models in the polycross progeny test, and (5) compare the accuracy of GS models from polycross versus full-sib progeny tests, as well as the genetic gains that can be obtained from both designs.

## Materials and methods

### Polycross progeny tests

In 1995, white spruce polycross progeny tests were initiated and replicated in several regions in the province of Quebec, Canada, to estimate the general combining ability of parental material forming the first-generation breeding population. These parent trees had been previously selected in provenance trials established in the 1950s and 1960s on various sites representative of the ecological conditions corresponding to white spruce reforestation zones in Quebec. Crosses for the next cycle were carried out using an equal volume of the pollen of the 19 males implicated in the polymix.

**Fig. 1:**
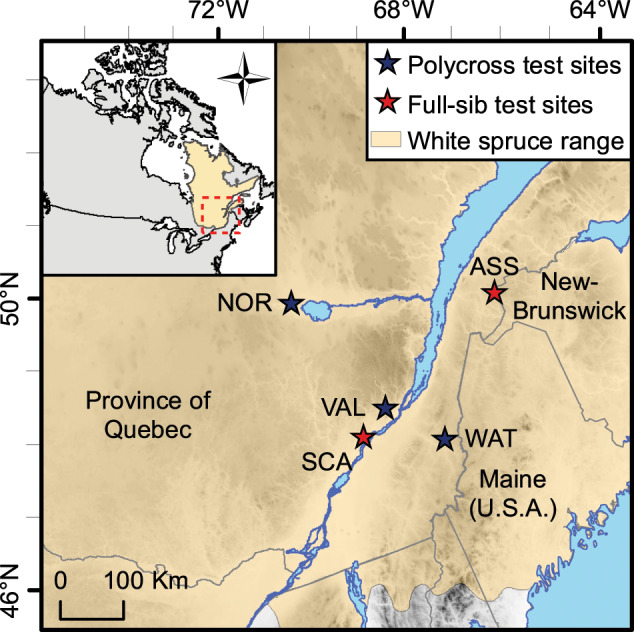
Locations of the polycross and full-sib progeny test sites in the province of Quebec, Canada. Polycross progeny test sites are Normandin (NOR), Valcartier (VAL), and Watford Township (WAT). Full-sib progeny test sites are Asselin (ASS) and St. Casimir (SCA).

Cold-stratified seeds were sown in February 1995 and seedlings were raised in a greenhouse at the Laurentian Forestry Centre (City of Québec, Canada) until June 1995 and then moved to the Valcartier Forest Experiment Station nursery (Lat. 46^o^ 55′ N, Long. 71^o^ 30′ W, Elev. 208 m) until plantation. For the present study, trees were sampled from three polycross progeny test sites: the first one in Valcartier (site VAL, Lat. 46^o^ 58′ N, Long. 71^o^ 28′ W, Elev. 212 m, planted in 1996) located in the sugar maple-basswood bioclimatic domain, the second in Normandin (site NOR, Lat. 48^o^ 50′ N, Long. 72^o^ 31′ W, Elev. 122 m, planted in 1997) located in the balsam fir-yellow birch bioclimatic domain, and the third in the Watford Township (site WAT, Lat. 46^o^ 15′ N, Long. 70^o^ 34′ W, Elev. 300 m, planted in 1997) located in the sugar maple-yellow birch bioclimatic domain (Fig. 1). The experimental layout followed a randomized complete block design with four blocks. In each block, four single-tree plots per family were arranged in an interlocking layout to allow for systematic thinning (Libby and Cockerham 1980). The initial spacing between trees was 2 m × 2 m at the NOR and WAT sites, and 1.5 m × 1.8 m at VAL due to space limitations.

Thirty-eight polycross families obtained with the same polymix were sampled for the present study (Table S1). Among the 19 males forming the polymix, three were also crossed as female parent, for a total of 54 genetically distinct parents involved in the polycross progeny test. Phenotypic traits were assessed on six to nine trees per family and per site, for a total of 892 trees (average of 23.5 trees per family). Tree height, DBH, and acoustic velocity were measured at age 19 since planting (i.e. measured in 2015 at VAL and in 2016 at NOR and WAT), while average wood density was assessed at age 18 since plantation. Total volume without bark (dm^3^) was calculated from height (m) and DBH (cm) following Prégent et al. (2010):

1 $$\mathrm{Volume} = 0.0344\,\left( {\mathrm{DBH}^{1.8329}} \right)\left( {\mathrm{Height}^{1.1793}} \right).$$

Acoustic velocity, which is a good proxy for mechanical wood stiffness, was measured on standing trees (Lenz et al. 2013). To obtain average wood density estimates, wood increment cores were collected from the south facing side of each tree. Cores were stored in a freezer, conditioned to 7% moisture content and cut to 1.68 mm thickness prior to X-ray densitometry analyses (Quintek Measurement Systems, TN, USA). Wood density was calculated as a ring area weighted mean from recorded pith to bark wood density profiles (Lenz et al. 2017).

### Full-sib progeny test

To compare GS accuracies obtained from the polycross mating design with those obtained from a disconnected partial diallel mating design, we also sampled offspring of the same parent trees in a full-sib progeny test replicated on two sites in Quebec. Full-sib crosses were conducted within each of six distinct breeding groups (Beaulieu 1996), which were designed to limit future inbreeding to within groups and control inbreeding build-up in the production population. Each parent was used in crosses from one to five times, giving rise to a mixture of full- and half-sib families within breeding groups. The two genetic test sites were established in 1999 from 2-year-old nursery-grown seedlings in the Asselin Township (site ASS, Lat. 47^o^ 55′ N, Long. 68^o^ 26′ W, Elev. 278 m) located in the balsam fir-yellow birch bioclimatic domain, and in St. Casimir (site SCA, Lat. 46^o^ 42′ N, Long. 72^o^ 06′ W, Elev. 52 m) located in the sugar maple-basswood bioclimatic domain (Fig. 1), respectively. The experiment layout was a randomized complete block design with ten replications. Trees were assigned to row-plots of five trees per plot (2 × 2 m spacing).

For the present study, a total of 1513 trees were sampled (732 from site ASS and 781 from site SCA) from 54 full-sib families involving 42 parents (Fig. S1, Table S1). These families were selected such that the mother, father, or both parents were among the 38 female parents retained for the polycross progeny test and such that full-sib and polycross progeny tests had highly similar genetic background. Thus, only four out of these 42 parents were not part of the polycross progeny test. The 54 full-sib families belonged to three different breeding groups. These 1513 trees were phenotyped for height, DBH, average density, and acoustic velocity at age 16 since plantation following the same procedures as for the polycross progeny test, and volume was estimated as above with Eq. (1).

Table 1 provides the summary statistics for the traits assessed in the polycross and full-sib progeny tests. Violin plots show the phenotypic distributions on each site (polycross, Fig. S2; full-sib, Fig. S3).

**Table 1:** Age after plantation at trait assessment time, phenotypic means, standard deviations (SD), and coefficients of variation (CV) across sites for the trees retained in analyses after pedigree verification for the polycross and full-sib progeny tests.

Trait^a^	Polycross dataset (*n* = 856)				Full-sib dataset (*n* = 1513)			
	Age	Mean	SD	CV (%)	Age	Mean	SD	CV (%)
Height (cm)	19	941.4	192.8	20.5	16	775.5	112.4	14.5
DBH (mm)	19	132.4	30.4	23.0	16	114.6	19.8	17.3
Volume (dm^3^)	19	61.2	35.6	58.2	16	35.8	15.5	43.4
Acoustic velocity (km/s)	19	3.3	0.5	13.8	16	3.0	0.5	16.3
Wood density (kg/m³)	18	374.1	29.0	7.8	16	366.7	32.3	8.8

^a^Measured traits in descending order are tree height, diameter at breast height, total volume without bark, acoustic velocity, and average wood density

### Genotyping assay

DNA samples for genotyping were isolated from needles and terminal buds by using the Qiagen DNeasy Plant Kit (Mississauga, ON, Canada) and quantified using the PicoGreen fluorescent dye (Thermo Fisher Scientific, Waltham, MA). For genotyping purposes, an Infinium iSelect SNP array (Illumina, San Diego, CA) containing 5308 correctly manufactured type II SNPs (one bead per SNP) was used (Appendix 1). The array was a subset of successful SNPs from a previous white spruce Infinium iSelect genotyping array (PgLM3) developed for linkage mapping and GS applications in white spruce (Pavy et al. 2013a; Beaulieu et al. 2014). To maximize genome coverage, one SNP per gene locus was used, with genes described in the annotated catalogue of white spruce expressed genes (Rigault et al. 2011). The 5308 gene loci underlying these SNPs were distributed across the 12 white spruce linkage groups representing all spruce chromosomes (Pavy et al. 2017). Genotyping was conducted by Neogene Canada (Edmonton, Alberta, Canada). Using two control samples per genotyping plate, the reproducibility rate of the assay was estimated at 99.99%.

Of the 5308 correctly manufactured SNPs, 5252 were deemed valid on the first set of 892 trees from the polycross population: 56 SNPs were discarded because they had low or null signal, or average call rate < 85%, or minimum allele frequency (MAF) <0.01, or potentially erroneous/paralogous SNPs with absolute value of fixation index |*F*_*e*_| ≥ 0.50. Valid SNPs had an average call rate of 99.8%, average MAF of 0.222, and average *F*_*e*_ of 0.020. For the second set of 1513 full-sib progeny trees, 4661 SNPs were deemed valid following the same criteria as above. Valid SNPs had an average call rate of 99.6%, average MAF of 0.222, and average *F*_*e*_ of −0.019. Less SNPs were retained for the population of full-sibs due to DNA samples being in suboptimal concentration for a subset of trees.

For GS modelling purposes (see below) using high quality SNPs for both full-sib and polycross data, a restricted set of 4092 SNPs was delimited from the set of 4661 SNPs described above, following the verification of Mendelian segregation of each SNP using 2700 progeny trees from a larger set of 80 full-sib crosses for which we had parent genotypes for the full set of 5308 correctly manufactured SNPs. SNPs with ≥5% of genotyping errors, that is, incompatible offspring genotypes according to parent genotypes, or for which offspring genotype frequencies departed significantly from Mendelian expectations in more than 25% of families (Fisher exact tests, *p* value < 0.05) were discarded. After all above SNP quality control filters, all retained trees in polycross and full-sib datasets had call rates ≥87%, with 99.5% of the trees having call rates ≥95% (polycross: mean call rate = 99.9%; full-sib: mean call rate = 99.6%). Rare missing genotypes (polycross, 0.2% of genotypes; full-sib, 0.4%) were imputed using a *k*-nearest neighbour method based on linkage disequilibrium (LD-kNNi) with the software LinkImpute (Money et al. 2015) for the polycross and full-sib progeny datasets separately. The software estimated an accuracy of 0.79 and 0.87 of imputed genotypes for the polycross and full-sib progeny datasets, respectively, by randomly masking of 10,000 genotypes.

For parentage assignments in the polycross progeny test, 46 out of the 54 parents were genotyped on a subset of 656 SNPs, with DNA samples not being available for eight of the 19 original pollen donors. Genotypes of all parents implicated in the full-sib crosses were available for the same 656 SNPs.

### Paternity recovery for the polycross progeny test

For the polycross progeny test, we verified the identity of the female parents and assigned paternity by using the two software COLONY v.2.0.6.3 (Wang and Santure 2009; Jones and Wang 2010) and CERVUS v.3.0 (Marshall et al. 1998; Kalinowski et al. 2007). With both methods, female and male parents of the 892 polycross offspring were considered unknown and the 46 genotyped parents as candidate parents. CERVUS is a pairwise likelihood comparison approach and calculates likelihood scores for each candidate parent-offspring pairs. In CERVUS, we ran the “parent pair-sexes unknown” analysis to find the most likely female and male parents for each offspring. The female parent of each offspring was confirmed when the LOD score was positive and there was fewer than 2.5% of allele mismatch. For paternal assignments, we determined the significance by using the delta score, which is the difference in LOD scores between the most likely candidate parent pair and the second most likely candidate parent pair. The critical delta score to assign parentage with 95% confidence was determined by simulating 10,000 offspring and assuming a genotyping error rate of 2% and that 50% of candidate parents were sampled, thus allowing for the missing eight paternal genotypes and possible pollen contamination. In addition, the LOD score of the male parent had to be positive for successful paternity assignments.

As for COLONY, it is a full pedigree likelihood method that infers sibship and parentage among individuals by searching for the configuration with the maximum likelihood. It allows for the clustering of offspring into maternal and paternal families even when parents are not genotyped. Hence, COLONY was particularly useful to infer the paternal families from the eight pollen donors in the polymix that could not be genotyped. COLONY was run with the default options and with a weak prior for maternal and paternal sibship size of 40 each. Maternal and paternal sibships were inferred from the best pedigree configuration with the maximum likelihood score. We verified that the inferred maternal and paternal sibships were identical between two independent runs with different random seed numbers.

After discarding 11 offspring that could not be assigned to their expected mother by COLONY and CERVUS, 23 other offspring resulting from pollen contamination (see results), and 2 outliers for tree height and acoustic velocity after checking GS model residuals (see below), a total of 856 trees were kept for further analyses of the polycross progeny test data.

For the full-sib progeny tests, the same procedure using COLONY and CERVUS using the 656 SNPs genotyped for each parent was previously conducted for another study (unpublished), and offspring that did not belong to their expected full-sib cross were discarded so that no pedigree errors remained for the final set of 1513 full-sib offspring retained for GS analyses. Genetic diversity of the polycross and full-sib progeny tests was estimated with the status number (*Ns* reported in Table S1):

2 $$Ns = 1/2\theta,$$

where *θ* is the group coancestry estimated from the reconstructed full pedigree (Lindgren et al. 1996).

### Conventional pedigree-based models and GS models

All analyses were conducted separately for the polycross and full-sib progeny tests in the R v.3.3.1 environment (R Core Team 2016). For the polycross progeny test, the predictive accuracy of breeding values obtained from GS models using the realized genomic relationships between trees (GBLUP) was compared with that obtained from the conventional pedigree-based method (ABLUP). The pedigree-based additive relationship matrix (***A***) was computed using the function “asreml.Ainverse” of the R package ASReml-R v.3.0 (Butler et al. 2007). Two pedigree-based models (ABLUP method) were tested using two different ***A*** matrices (1) the matrix computed from the partial polycross pedigree with known mothers, but unknown fathers (ABLUP partial pedigree); and (2) the matrix computed from the full polycross pedigree with known mothers and retrieved fathers using the paternity assignment analysis (ABLUP full pedigree). For the GBLUP method, the realized additive genomic relationship matrix (***G***) and its inverse were computed from the marker data using the “A.mat” function of the R package rrBLUP (Endelman and Jannink 2012) with the default options, which was equivalent to the formula described by VanRaden (2008). The resulting matrix for offspring and the pairwise relatedness values between offspring within maternal families and recovered paternal families are shown in Figs. S4 and S5 respectively.

For the full-sib progeny test, we only tested the GBLUP method since our goal was to compare the genomic prediction accuracy obtained using the partial diallel mating design with that obtained from the polycross design. The resulting matrix and the pairwise relatedness values between offspring within full-sib families are shown in Figs. S6 and S7, respectively. To make the sample size comparable with that of the polycross progeny test, we randomly sampled 856 individuals among the 1513 available offspring, and then ran the GBLUP models to calculate the desired genetic parameters (see below). This sample size corresponded to about eight trees per full-sib family per site, which was similar to the number of trees per polycross family per site. We repeated this process ten times and averaged the estimated genetic parameters.

The polycross and the full-sib datasets were analyzed separately by fitting individual-tree mixed models (the so-called “animal model”) in ASReml-R v.3.0 of the form:

3 $${\boldsymbol{y}} = {\boldsymbol{X}}{\boldsymbol{\beta }} + {\boldsymbol{Z}}_1{\boldsymbol{a}} + {\boldsymbol{Z}}_2{\boldsymbol{sa}} + {\boldsymbol{e}},$$

where ***y*** is the phenotype; ***β*** represents vectors of fixed effects including the overall mean, the breeding group (full-sib test), the site, and the block within site; ***a*** is the random additive genetic effect, with ${\boldsymbol{a\sim }}N\left( {{\mathbf{0}},\sigma _a^2{\boldsymbol{A}}} \right)$ for the ABLUP method and ${\boldsymbol{a}}\sim N\left( {{\mathbf{0}},\sigma _a^2{\boldsymbol{G}}} \right)$ for the GBLUP method; ***sa*** is the random interaction of site with additive genetic effects, with ${\boldsymbol{sa}}\sim N\left( {{\mathbf{0}},\sigma _{sa}^2{\boldsymbol{I}}_{\boldsymbol{s}}\,\otimes\,{\boldsymbol{A}}} \right)$ for the ABLUP method and ${\boldsymbol{sa}}\sim N\left( {{\mathbf{0}},\sigma _{sa}^2{\boldsymbol{I}}_{\boldsymbol{s}}\,\otimes\,{\boldsymbol{G}}} \right)$ for the GBLUP method; and ***e*** is the residual term, with ${\boldsymbol{e}}\sim N\left( {{\mathbf{0}},\sigma _e^2{\boldsymbol{I}}_{\boldsymbol{e}}} \right)$. The ***X*** and ***Z*** matrices are incidence matrices of their corresponding effects, and ***I***_*x*_ is an identity matrix of its proper dimension. The symbol ⊗ refers to the Kronecker product. To test the hypothesis of greater than zero variance for each random effect (*H*_0_: *σ*^2^ = 0; *H*_1_: *σ*^2^ > 0), we performed a likelihood ratio test with one degree of freedom between the full model in Eq. (3) and a reduced model without the effect to be tested. Individual narrow-sense heritability ($\widehat h_{ind}^2$) was estimated as:

4 $$\widehat h_{ind}^2 = \widehat \sigma _a^2/\left( {\widehat \sigma _a^2 + \widehat \sigma _{sa}^2 + \widehat \sigma _e^2} \right)$$

The amplitude of genotype-by-environment interaction (G × E), or type-B correlation ($\widehat r_B$), was estimated by

5 $$\widehat r_B = \widehat \sigma _a^2/\left( {\widehat \sigma _a^2 + \widehat \sigma _{sa}^2} \right)$$

Standard errors of heritability and type-B correlation estimates were obtained using the delta method (pin function from the R package nadiv; Wolak 2012).

For all models, estimated breeding values (EBVs for ABLUP; GEBVs for GBLUP) of parents and offspring were obtained from the best linear unbiased predictions (BLUPs) of the random additive effect (***a***). The theoretical accuracy ($\widehat r$) of estimated parental and offspring breeding values was estimated as

6 $$\widehat r = \sqrt {1 - SE^2/\left( {\left( {1 + F_i} \right)\widehat \sigma _a^2} \right)},$$

where *SE* is the standard error of the breeding value obtained from Eq. (3), and *F*_*i*_ is the inbreeding coefficient of the *i*^th^ individual (Gilmour et al. 2015). The value of (1 + *F*_*i*_) was obtained from the diagonal elements of the *G* matrix. For the calculation of the theoretical accuracy of EBVs and make it comparable across models (i.e. ABLUP partial pedigree, ABLUP full pedigree, and GBLUP), we fixed the variance parameters $\sigma _a^2$ and $\sigma _{sa}^2$ in Eq. (3) to those estimated with the GBLUP method (El-Kassaby et al. 2011).

To determine whether dominance effects could be detected using the polycross progeny test, we fitted dominance effects using the realized full-sib crosses effect in the polycross (ABLUP full pedigree) or using the dominance genomic relationship matrix (GBLUP; Vitezica et al. 2013). For the full-sib progeny test, we fitted dominance using the dominance relationship matrix (GBLUP). The dominance models are described in Appendix 2.

### Cross-validation

To further assess the accuracy of offspring’s breeding values resulting from the polycross and full-sib progeny testing, we performed a tenfold cross-validation (CV) scheme combining data across sites (Beaulieu et al. 2014; Lenz et al. 2017, 2020). CV was performed separately for the polycross and full-sib progeny tests. For CV, offspring were randomly split into tenfold, each containing ~10% of the trees from each family. For each round of CV, ninefold were used in model training (Eq. (3)), which was used to predict the breeding values for the remaining fold. This tenfold CV was repeated ten times, for a total of 100 models for each trait. For the full-sib progeny test, we randomly sampled 856 individuals among the 1513 available offspring for each of the ten repetitions of tenfold.

The predictive ability (PA) of the models was evaluated as the Pearson correlation coefficient between the predicted breeding values of the validation trees and adjusted phenotypes (y*). Adjusted phenotypes were obtained by taking the residuals (y* = ***e***) of a model that included an overall mean and the fixed effects (***β***) of the breeding group (full-sib test), the site, and the block within site: ***y*** = ***Xβ*** **+** ***e***. The predictive accuracy (PACC) of models was estimated from PA as $PACC = PA/\sqrt {\widehat h_{ind}^2}$ (Dekkers 2007; Legarra et al. 2008). For both ABLUP and GBLUP models, we used the estimated $\widehat h_{ind}^2$ from the GBLUP model as our best estimate of narrow-sense heritability. Therefore, the comparison of PACC between methods is not affected by the heritability estimates and depends only on differences in PA. PA and PACC were calculated across folds by combining the predicted breeding values of validation trees from the tenfold and then averaged across repetitions to obtain standard errors.

### Expected genetic gains

Expected genetic gains were calculated as the mean of the top 5% predicted genomic-estimated breeding values (mean GEBVs) of the validation trees (i.e. prediction without phenotypes) as estimated from the CV procedure of the GBLUP analysis in Eq. (3) and for each trait separately. The gains for each trait were averaged across the ten repetitions in CV. To compare results between the polycross and full-sib progeny tests, we multiplied the expected genetic gains by the predictive accuracy (PACC) obtained from CV. Finally, we calculated the expected genetic gains per year by assuming that GS implemented with a full-sib mating design requires 9 years for the deployment of the next generation material, that is 4 years for crosses and production of seedlots, at most 1 year (6 months with available genotyping chip) for genotyping and predicting breeding values with GS models, and 4 years for vegetative propagation of selected individuals for seedling production (Lenz et al. 2017). For the polycross mating design, we assumed that conducting crosses requires only 2 years, for a total of 7 years before deployment, due to the easier handling of the pollen mix and because pollen banks are already available for the Quebec white spruce breeding program. For the polycross progeny test presented in this study, all crosses could be realized in a single year, and thus we were conservative by assuming 2 years for conducting these crosses.

## Results

### Paternity recovery for the polycross progeny tests

**Fig. 2:**
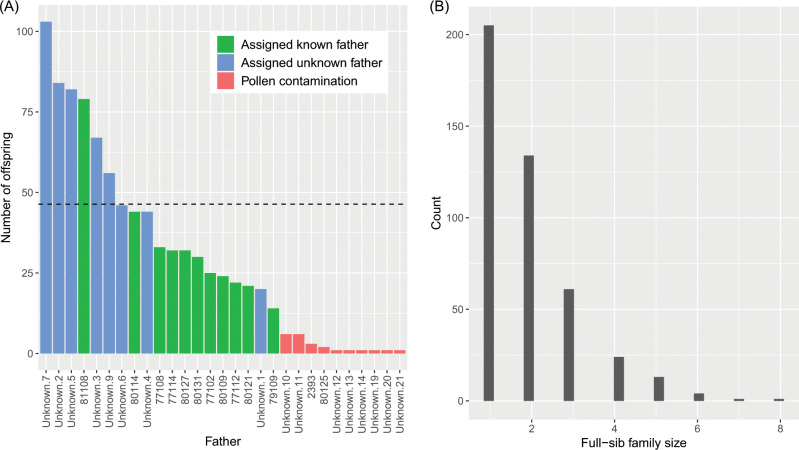
**a** Number of offspring per pollen donor for the polycross progeny test. Fathers labelled as “unknown” on the *x*-axis represent paternal families from non-genotyped fathers that were inferred by the software COLONY. The dashed line represents the expected equal male reproductive success of 46.4 offspring per male parent. **b** Histogram of full-sib family size (i.e. offspring sharing the same mother and father) for the polycross progeny test.

We first verified the identity of each maternal parent and assigned paternity using the software CERVUS and COLONY. Eleven offspring (1.2%) could not be assigned to any of the 38 candidate mothers using both software. The two software fully agreed on maternal assignments for the remaining 881 offspring. CERVUS and COLONY also fully agreed for paternity assignments of 361 offspring (41%) to the 11 known genotyped candidate paternal trees forming the polymix (Fig. 2a, eight fathers could not be genotyped). Unexpectedly, five offspring were assigned paternity to trees that were only crossed as maternal parents in the polycross mating design (parents 2393 and 80125 in Fig. 2a).

The remaining 520 offspring (59%) could not be assigned to candidate parents by CERVUS (LOD scores are shown in Fig. S8), but clustered into paternal families from unknown fathers in COLONY. The number of offspring assigned to each of the unknown fathers was the same across independent runs of COLONY, indicating that the model converged. Eight unknown fathers contributed to 20 or more offspring, which corresponded to the eight pollen donors in the polymix that were not genotyped (Fig. 2a). All other unknown paternal families included six offspring or less and were considered to originate from pollen contamination (total of 23 offspring, 2.6%). Only one foreign father (unknown 11) contaminated offspring in four different maternal families, while the other nine foreign pollen donors had progeny in a single maternal family each. One particular maternal family (2254) was more severely affected by pollen contamination as 12 out of 23 offspring were sired by six foreign pollen donors, while for all other contaminated maternal families, one to three offspring were sired by a single foreign pollen donor in each family.

After discarding the 23 offspring resulting from pollen contamination, the contributions of the 19 pollen donors in the polymix were found to be highly variable (Fig. 2a). The eight non-genotyped fathers had on average a higher reproductive success than the 11 genotyped fathers. The number of offspring sired by each male parent strongly departed from the expected equal male reproductive success of 46.3 offspring per father (chi-squared = 271.4, *p* value < 2.2e–16). The maternal family sizes varied from 11 to 25 (mean = 22.5) and the paternal family sizes varied from 14 to 103 (mean = 45.1). The polycross resulted in 443 realized biparental crosses and small full-sib family sizes (mean = 1.9, median = 2 offspring per full-sib family; Fig. 2b). Mothers were crossed with 4–15 fathers (mean = 11.7), and fathers were crossed with 10–35 mothers (mean = 23.3).

### Genomic prediction versus conventional pedigree-based methods for the polycross progeny test

We compared the performance of GS models (GBLUP method) with the conventional pedigree-based models using either the partial pedigree with known mothers only (partial pedigree ABLUP model) or using the fully reconstructed pedigree with recovered fathers (full pedigree ABLUP model). Using ABLUP with the partial pedigree, the wood quality traits acoustic velocity and average wood density were the most heritable ($\widehat h_{ind}^2$ = 0.48 and $\widehat h_{ind}^2$ = 0.42, respectively), while the growth traits height, DBH, and volume harboured moderate heritability ($\widehat h_{ind}^2$ from 0.27 to 0.32; Table 2, variance components are reported in Table S2). Wood quality traits showed high type-B genetic correlations ($\widehat r_B$ = 0.93 for both traits), indicating very low genotype-by-environment interactions (G × E), while growth traits showed higher G × E although still low and not significant ($\widehat r_B$ from 0.72 to 0.81). We found similar trends using the full pedigree ABLUP and the GS models (GBLUP).

**Table 2:** Estimates of individual narrow-sense heritability ($\widehat h_{ind}^2$) and type-B genetic correlation ($\widehat r_B$) for the polycross and full-sib progeny tests.

Trait^a^	Model^b^	$\widehat h_{ind}^2$	$\widehat r_B$
**Polycross progeny test (*n*** **=** **856 trees)**			
Height	ABLUP partial pedigree	0.30 (0.11)***	0.72 (0.21)
	ABLUP full pedigree	0.21 (0.07)***	0.64 (0.17)*
	**GBLUP**	**0.20** (0.06)***	**0.60** (0.16)**
DBH	ABLUP partial pedigree	0.27 (0.11)***	0.81 (0.25)
	ABLUP full pedigree	0.23 (0.07)***	0.74 (0.17)
	**GBLUP**	**0.21** (0.06)***	**0.70** (**0.17)**
Volume	ABLUP partial pedigree	0.32 ((0.12)***	0.73 (0.20)
	ABLUP full pedigree	0.25 (0.08)***	0.72 (0.16)*
	**GBLUP**	**0.22** (0.06)***	**0.65** (0.15)*
Acoustic velocity	ABLUP partial pedigree	0.48 (0.14)***	0.93 (0.16)
	ABLUP full pedigree	0.44 (0.09)***	0.91 (0.10)
	**GBLUP**	**0.41** (0.06)***	**0.92** (**0.09)**
Wood density	ABLUP partial pedigree	0.42 (0.13)***	0.93 (0.19)
	ABLUP full pedigree	0.42 (0.09)***	1.00 (0.00)
	**GBLUP**	**0.37** (0.06)***	**1.00** (**0.00)**
**Full-sib progeny test (10 random samples of** ***n*** **=** **856 trees out of 1513)**			
Height	**GBLUP**	**0.18** (0.07)*	**0.67** (0.19)*
DBH	**GBLUP**	**0.06** (**0.05)**	**0.39** (0.33)*
Volume	**GBLUP**	**0.11** (**0.06)**	**0.53** (0.26)*
Acoustic velocity	**GBLUP**	**0.35** (0.07)***	**0.89** (**0.09)**
Wood density	**GBLUP**	**0.36** (0.07)***	**0.86** (**0.10)**

The models tested are: “ABLUP partial pedigree” is the conventional pedigree-based model using the partial polycross pedigree with known mothers, but unknown fathers; “ABLUP full pedigree” is the model using the the full polycross pedigree with known mothers and retrieved fathers; “GBLUP” is the genomic selection model using the realized additive genomic relationship matrix (***G***) with results in bold type to facilitate comparisons between the polycross and full-sib progeny tests. Standard errors of estimates are in parentheses. For heritability, the significance of the additive variance component is shown (see Tables S2 and S4)^c^. For type-B genetic correlations, the significance of the site x additive variance component is shown^c^. A significant site x additive variance indicates a significant genotype-by-environment interaction (i.e. smaller values of $\widehat r_B$)

^a^See Table 1 for a full description of traits

^b^The model fitted is described in Eq. (3) in the manuscript

^c^Level of statistical significance: *P < 0.05; **P < 0.01; ***P < 0.001

For all traits, the heritabilities estimated by the full pedigree ABLUP or by the GBLUP model decreased and were more precise, as indicated by smaller standard errors compared with ABLUP using the partial pedigree (Table 2). The GBLUP model resulted in the smallest heritability estimates associated with the smallest standard errors for all traits. Similarly, $\widehat r_B$ decreased (indicating higher G × E) for growth traits when using the GBLUP model as compared with both ABLUP models, but $\widehat r_B$ for wood quality traits remained very high (indicating low G × E) for all three models. We did not detect significant dominance effects for any of the traits (Table S3).

We calculated two different estimates of predictive accuracy: (1) the theoretical accuracy that is based on the standard errors of breeding values; and (2) the predictive accuracy of breeding values (PACC) that is based on repetitive prediction of offspring’s breeding values in cross validation (CV). The average theoretical accuracy of parental breeding values for the 38 maternal trees was high for growth traits (from 0.71 to 0.73 using ABLUP with the partial pedigree) and was even higher for wood quality traits (0.83–0.84) (Table 3). The theoretical accuracy of parents’ breeding values obtained from the GBLUP model was lower than that obtained using both ABLUP methods. In contrast, the theoretical accuracy of offspring’s breeding values increased by 0.06–0.08 when using the full pedigree as compared with the partial pedigree, and further increased by another 0.03–0.04 with the GS (GBLUP) model.

**Table 3:** Estimates of theoretical accuracy of parents’ and offspring’s breeding values, predictive ability (PA) and predictive accuracy (PACC) of offspring’s breeding values obtained from cross-validation for the polycross and full-sib tests.

		Theoretical accuracy		Cross-validation	
Trait^a^	Model^b^	Parents’ BVs^c^	Offspring’s BVs	PA offspring’s BVs	PACC offspring’s BVs
**Polycross progeny test (*n*** **=** **856 trees)**					
Height	ABLUP partial pedigree	0.71 (0.02)	0.51 (0.04)	0.23 (0.01)	0.51 (0.02)
	ABLUP full pedigree	0.72 (0.03)	0.59 (0.03)	0.26 (0.01)	0.58 (0.03)
	**GBLUP**	**0.68** (**0.04)**	**0.63** (**0.01)**	**0.28** (**0.01)**	**0.62** (**0.03)**
DBH	ABLUP partial pedigree	0.72 (0.03)	0.52 (0.04)	0.20 (0.01)	0.45 (0.01)
	ABLUP full pedigree	0.73 (0.04)	0.61 (0.03)	0.26 (0.01)	0.58 (0.02)
	**GBLUP**	**0.69** (**0.04)**	**0.65** (**0.01)**	**0.28** (**0.01)**	**0.61** (**0.02)**
Volume	ABLUP partial pedigree	0.73 (0.02)	0.53 (0.04)	0.24 (0.01)	0.51 (0.01)
	ABLUP full pedigree	0.74 (0.03)	0.61 (0.03)	0.28 (0.01)	0.59 (0.01)
	**GBLUP**	**0.70** (**0.04)**	**0.65** (**0.01)**	**0.29** (**0.01)**	**0.62** (**0.02)**
Acoustic velocity	ABLUP partial pedigree	0.84 (0.02)	0.67 (0.03)	0.29 (0.01)	0.46 (0.01)
	ABLUP full pedigree	0.85 (0.03)	0.74 (0.02)	0.43 (0.00)	0.66 (0.01)
	**GBLUP**	**0.81** (**0.04)**	**0.77** (**0.01)**	**0.45** (**0.01)**	**0.70** (**0.01)**
Wood density	ABLUP partial pedigree	0.83 (0.02)	0.66 (0.03)	0.27 (0.01)	0.44 (0.01)
	ABLUP full pedigree	0.85 (0.03)	0.72 (0.02)	0.36 (0.01)	0.59 (0.01)
	**GBLUP**	**0.80** (**0.04)**	**0.76** (**0.01)**	**0.39** (**0.01)**	**0.63** (**0.01)**
**Full-sib progeny test (10 random samples of** ***n*** **=** **856 trees out of 1513)**					
Height	**GBLUP**	**0.66** (**0.06)**	**0.62** (**0.02)**	**0.28** (**0.04)**	**0.66** (**0.04)**
DBH	**GBLUP**	**0.48** (**0.06)**	**0.44** (**0.02)**	**0.14** (**0.05)**	**0.61** (**0.11)**
Volume	**GBLUP**	**0.59** (**0.06)**	**0.55** (**0.02)**	**0.20** (**0.04)**	**0.61** (**0.04)**
Acoustic velocity	**GBLUP**	**0.74** (**0.06)**	**0.71** (**0.02)**	**0.42** (**0.03)**	**0.71** (**0.03)**
Wood density	**GBLUP**	**0.74** (**0.06)**	**0.71** (**0.02)**	**0.44** (**0.03)**	**0.74** (**0.02)**

The models tested are: “ABLUP partial pedigree” is the conventional pedigree-based model using the partial polycross pedigree with known mothers, but unknown fathers; “ABLUP full pedigree” is the model using the full polycross pedigree with known mothers and retrieved fathers; “GBLUP” is the genomic selection model using the realized additive genomic relationship matrix (***G***) with results are in bold type to facilitate comparisons between the polycross and full-sib progeny tests. Standard errors of estimates are in parentheses

^a^See Table 1 for a full description of traits

^b^The model fitted is described in Eq. (3) in the manuscript

^c^Theoretical accuracy of breeding values for the 38 parents shared between the polycross and full-sib progeny tests (corresponding to the 38 females in the polycross progeny test)

The PA, which is the correlation between the predicted breeding values and the adjusted phenotypes in CVs, ranged from 0.20 for DBH to 0.29 for acoustic velocity using the partial pedigree ABLUP model (Table 3). PA increased by 0.03–0.14 when using ABLUP with the full pedigree as compared with the partial pedigree, particularly for acoustic velocity and wood density. For all traits, the GBLUP model had a small advantage as PA increased by 0.01–0.03 over the full pedigree ABLUP model. For both ABLUP and GBLUP models, PA was strongly related to heritability. Therefore, we standardized PA by dividing it by the square root of heritability to obtain estimates of predictive accuracy (PACC). For all three models, we used the same heritability estimates obtained from the GBLUP model so that methods could be compared on equal ground. Therefore, differences in PACC among methods are only due to differences in PA. The PACC of the GBLUP models were moderate to high (0.61–0.70). The PACC greatly increased by 0.11–0.16 for growth traits, and by 0.19–0.24 for wood quality traits when using GS (GBLUP) instead of ABLUP with the partial pedigree models.

### Prediction accuracies for the polycross versus the full-sib progeny test

We compared the accuracies obtained using GS models (GBLUP) for the polycross mating design to those obtained for the full-sib (partial diallel) mating design of the same parental breeding population and using the same training population size (*n* = 856). Heritability estimates ($\widehat h_{ind}^2$) obtained with GBLUP for height, acoustic velocity, and wood density were slightly higher in the polycross versus the full-sib progeny test, but they were not significantly different as shown by the overlapping standard errors (Table 2; variance components for the full-sib progeny test are in Table S4). For DBH and volume, $\widehat h_{ind}^2$ and $\widehat r_B$ estimates were much lower in the full-sib progeny test compared with the polycross progeny test. Similarly to the polycross mating design, we did not detect significant dominance effects in the full-sib mating design for any of the traits (Table S5).

The theoretical accuracy of parental breeding values (i.e. the 38 parents in common to both polymix and full-sib progeny test) and that of offspring’s breeding values were lower for the full-sib progeny test as compared with the polycross progeny test (GBLUP in Table 3). For DBH and volume, there was a large reduction in theoretical accuracy of parental and offspring’s breeding values in the full-sib progeny test, which was largely due to the low estimated additive genetic variance. The cross-validated predictive accuracy (PACC) of offspring’s breeding values was similar for all traits, except that it was larger for wood density in the full-sib versus the polycross progeny test. Care should be taken when comparing the PACC estimates for DBH and volume in the full-sib progeny test because of the small and non-significant heritability estimates for these traits, which can affect the precision of PACC estimates. For these two traits, PACC seemed to be overestimated in the full-sib progeny test, as shown by the much lower estimated values of theoretical accuracy.

### Genetic gains for the polycross versus the full-sib progeny test

We compared the expected genetic gains between the polycross and the full-sib progeny test after selecting the top 5% offspring (Table 4). Expected genetic gains varied from 5.9% for wood density to 22.5% for volume in the polycross progeny test, and from 3.3% for DBH to 13.2% for volume in the full-sib progeny test. It should be noted that the smaller genetic gains obtained for DBH and volume in the full-sib versus the polycross progeny test are due to a lower heritability, whereas heritability was higher and similar between both datasets for all other traits. Genetic gains for DBH and volume were therefore not comparable between the present polycross and full-sib progeny test. After correcting for differences in predictive accuracy (PACC), we found similar gains obtained for height, acoustic velocity, and wood density between the polycross and the full-sib progeny test. However, gains per year became higher for all traits for the polycross progeny test given that 7 years before deployment of selected material was assumed for this mating scheme as compared to 9 years for the full-sib mating design.

**Table 4:** Comparison of expected genetic gains and expected genetic gains per year derived from the polycross progeny test (*n* = 856 trees) and full-sib progeny test obtained from a partial diallel mating design (10 random samples of *n* = 856 trees out of 1513).

	*Ns* of selected trees^a^		Gain (%)		Corrected gain (%)^b^		Corrected gain per year (%/year)^c^	
Trait^d^	Polycross test	Full-sib test	Polycross test	Full-sib test	Polycross test	Full-sib test	Polycross test	Full-sib test
Height	11.85 (0.62)	5.88 (0.70)	6.36 (0.10)	5.69 (1.10)	3.92 (0.06)	3.71 (0.72)	0.56 (0.01)	0.41 (0.08)
DBH	9.43 (0.48)	7.60 (2.50)	9.35 (0.16)	3.26 (1.64)	5.67 (0.10)	2.04 (1.03)	0.81 (0.01)	0.23 (0.11)
Volume	9.79 (0.53)	5.47 (1.01)	22.53 (0.52)	13.24 (5.07)	14.04 (0.32)	7.93 (3.04)	2.01 (0.05)	0.88 (0.34)
Acoustic velocity	6.06 (0.21)	7.43 (0.91)	10.11 (0.12)	9.43 (0.72)	7.03 (0.09)	6.66 (0.51)	1.00 (0.01)	0.74 (0.06)
Wood density	10.94 (0.38)	5.55 (1.36)	5.94 (0.05)	6.16 (0.99)	3.75 (0.03)	4.43 (0.71)	0.54 (0.00)	0.49 (0.08)

Standard errors are in parentheses. Gains are expressed as percentage of the phenotypic mean

^a^*Ns* = Status number of selected trees calculated from Eq. (2)

^b^Expected genetic gain multiplied by the predictive accuracy (PACC) for each trait, as estimated from cross-validation

^c^(Corrected gain)/(time required for the deployment of the next generation material), that is 7 years for the polycross mating design and 9 years for the partial diallel mating design (see “Methods” for details)

^d^See Table 1 for a full description of traits

## Discussion

The increasing access to genetic markers at the genome-wide level presents a wealth of opportunities to tree breeders and is redefining selection strategies in long-standing conifer breeding programs. Notwithstanding recently published spruce genome sequences (for a review, De La Torre et al. 2014), large annotated genome-wide marker resources have been generated for different spruce species and breeding programs during the last 10 years (e.g. Pavy et al. 2008, 2013a, 2013b, 2016; Azaiez et al. 2018). As shown in the present study, these high quality resources have resulted in the design of reliable genotyping chips with high reproducibility rate and little missing data for the great majority of Mendelian markers.

The high accuracy of genomic prediction of offspring’s breeding values obtained for the present polycross progeny test emphasizes the opportunity to use this mating design in forward GS schemes. The opportunity to maintain genetic diversity by crossing more parents with diverse genetic backgrounds in a shorter period of time outcompeted the full-sib (partial diallel) mating design in terms of expected genetic gains. Therefore, GS based on polycross mating appears as a valuable approach to especially enhance the gain output of early cycles in conifer breeding.

### Paternal assignment and GS models allow for improved genetic parameter estimates for the polycross mating design

Using a relatively small set of 657 SNPs, we could recover the maternal and paternal contributions of polycross offspring with a high level of confidence. COLONY and CERVUS fully agreed on maternal assignments (881 offspring) as well as on paternal assignments when the father was genotyped (356 offspring). These results suggest a very high accuracy of assignments given that the two software work very differently: CERVUS is a pairwise likelihood method whereas COLONY is a full pedigree likelihood method that searches for the most likely sibship configuration. None of the software assigned the correct mother for a small portion of offspring (1.2%), which could be caused by miss-assignments, but also errors in handling of crosses or in field test establishment may occur in tree breeding operations (e.g. Godbout et al. 2017).

Some predicted pollen donors contributed to significantly more siblings than others. Unexpectedly, paternal families from non-genotyped “unknown” fathers were on average larger than paternal families from genotyped fathers. This could occur by chance, but could also be caused by miss-assigned offspring. However, such large family sizes are possible since the fourth largest family was from a known genotyped father. The average sibship probabilities were high for offspring assigned to both known and unknown fathers (Fig. S9). Furthermore, the average coefficient of relatedness within paternal families was around 0.25, thus in line with expectations (Fig. S5B). Only the largest paternal family (father unknown #7) showed some pairwise relatedness values closer to zero that could indicate the inclusion of a few unrelated individuals. Given these evidences, we trust that the reconstructed pedigree is mostly accurate, but may be slightly improved if we had genotypes for all candidate fathers. However, a very small number of miss-assigned individuals would only cause minor differences in predictive accuracy of the ABLUP full-pedigree model, and thus would not modify the general conclusions of this study.

Our observations of unequal paternal contributions are similar to previous studies, for example, in *Pseudotsuga menziesii* var. *menziesii* (El-Kassaby and Ritland 1992), *Picea rubens* (Doerksen and Herbinger 2008), *Cryptomeria japonica* (Moriguchi et al. 2009), and *Pinus pinaster* (Vidal et al. 2015). This is most likely due to the fact that the pollen mixture was assembled based on volume but not on germination success rate in the analysed polycross progeny test. Together with the fact that exact breeding values of males forming the polymix were unknown at the time of selection and could have deviated from the theoretical assumed neutral state, as observed for growth traits in the present polycross population (Fig. S10), genetic parameter estimates using the partial pedigree appear to be most likely biased. In the present analysis, heritability was likely overestimated in ABLUP analyses using the partial (i.e. maternal) pedigree. ABLUP models considering both maternal and paternal information and GBLUP models led to more conservative estimates of heritability carrying smaller standard errors than estimates obtained using the partial pedigree. Similar observations were made for type-B correlations for growth traits, indicating that significant G × E interaction may not be precisely detected with partial pedigree-based analyses. The overestimation of genetic control and non-detection of G × E interaction would severely impact genetic gain predictions and selection decisions for different environments or breeding zones. The test sites used in the present study were scattered across the two major breeding zones delimited for white spruce in the province of Quebec (Li et al. 1997), and the larger G × E noted herein for growth traits ($\widehat r_B$ from 0.60 to 0.70 using GBLUP) confirmed the need to consider these breeding zones in selection and deployment strategies.

### GS leads to improved prediction of offspring genetic merit for the polycross progeny test

Pedigree reconstruction in the polycross progeny test greatly improved the theoretical accuracies of offspring’s breeding values by 14–25% and the predictive accuracies (PACC) by 22–52% in CVs. Still, genomic prediction (GBLUP) increased theoretical accuracies values by an additional 4–7% and PACC values by another 5–7%. This trend may be due to the better estimation of Mendelian sampling in GS models by using the realized relationship matrix (Grattapaglia et al. 2018). Also, genetic differences between paternal alleles within maternal families may be better predicted using GS. However, the present polycross progeny dataset did not consider enough individuals per maternal family to precisely evaluate within-maternal-family accuracy. The precision of GS models could likely be further enhanced in larger sample sets and by estimating non-additive genetic components (Gamal El-Dien et al. 2016). Perhaps due to the relatively small sample size and small realized full-sib family size (Fig. 2b), we were not able to clearly determine the existence of dominance effects in the analysed polycross dataset. Future studies may show to which degree non-additive effects can be estimated in operational-size polycross progeny tests with larger full-sib family sizes.

Our results show that breeding strategies that rely on somewhat cheaper low density marker panels for pedigree reconstruction would also obtain high accuracies in forward selection using the ABLUP model. In this study, we used a small number of SNPs (656) for accurate pedigree reconstruction, while Vidal et al. (2015) performed pedigree reconstruction with even <100 well-chosen SNPs. If genotyping costs are a limiting factor, a smaller genotyping assay may be helpful to reconstruct the pedigree for a portion of the breeding population, which can then be integrated together with another set of genotyped trees on a denser marker panel into a single-step GS model (HBLUP, Legarra et al. 2009). However, as seen in the present study, additional precision can be obtained when all trees are genotyped for a few thousand SNPs.

In this study, we found similar estimates of predictive accuracies of offspring’s breeding values obtained from polycross and full-sib (partial diallel) mating and testing designs. Estimates of total genetic gain were also similar between both designs, except for DBH and volume. Nevertheless, genetic gain per unit time was higher for the polycross progeny test because crosses can be conducted more quickly and the time needed to deploy the selected material can thus be further shortened by about 2 years, compared with the partial diallel mating design. It is challenging to compare different mating designs based on empirical data because of the slightly different levels of genetic diversity, heritability, and predictive accuracy of breeding values. Using simulations and pedigree reconstruction, Bouffier et al. (2019) found lower total genetic gain of the polycross design compared with the full-sib double-pair mating design, but found higher gains per breeding cycle when they assumed that the polycross breeding cycle could be shortened by 1 year, thus corroborating our findings. In the present study, we compared two operational breeding designs set up for the white spruce breeding program in Quebec. The similar genetic background between the two datasets presents quite a unique opportunity to test hypotheses in real breeding conditions. The polycross allowed crossing more parents because the 19 fathers in the polymix were mostly not overlapping with the 38 female parents (only three parents in common), resulting in higher genetic diversity in the selection population for fewer crosses (38 polycrosses, *Ns* = 38.46) than the partial diallel mating design (54 full-sib crosses, 42 parents, *Ns* = 36.19). In the partial diallel, each parent must be crossed at least twice, and ideally four to six times (Lambeth et al. 2001), in order to estimate their general combining ability, thus reducing the total number of parents that can be crossed with equal breeding efforts compared with the polycross mating design. These differences inherent to these operational mating designs also resulted in smaller status number of the top 5% selections for the full-sib progeny test given that the selections originated from a few best full-sib families with common parentage (average of 9.1 full-sib families from 13.3 parents), as it is often the case for these types of design (Lambeth et al. 2001). Thus, adding a diversity constraint on the selected material would further reduce the genetic gains obtained in the full-sib progeny test and advantage the polycross mating design. Thus, our finding that the gain per year is greater for the polycross mating design should be conservative.

### Polycross mating integrates well into forward GS

In conifer breeding, polycross mating was traditionally used under a backward selection scheme to evaluate the general combining ability of female trees to be propagated in seed orchards or to plan further controlled crosses of superior genotypes to augment genetic gain. In the present study, the theoretical accuracy of parental breeding values indicated a small advantage of pedigree-based methods over GS in the case of backward selection, but the accuracy of breeding values in forward selection was higher for GS.

**Fig. 3:**
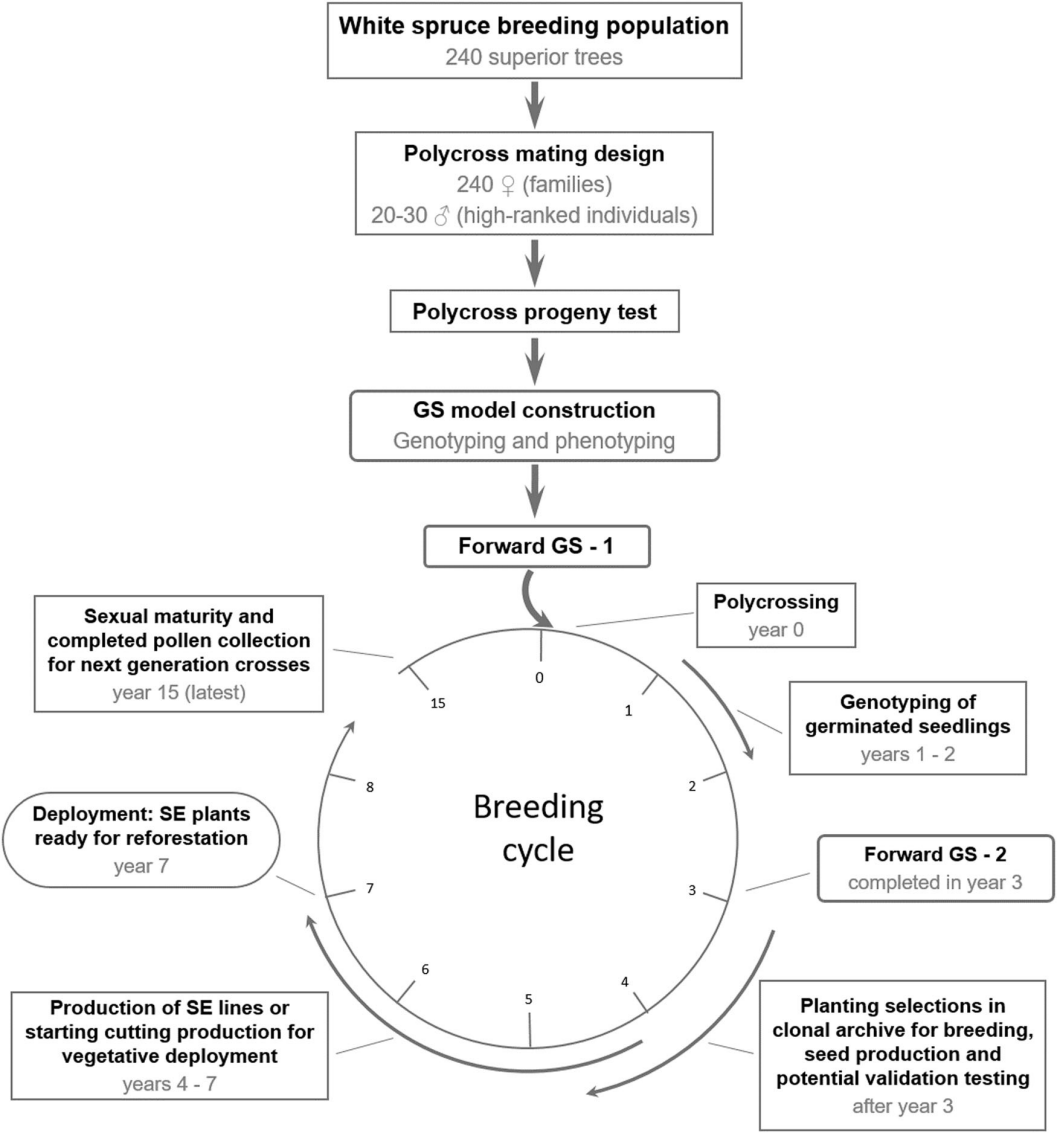
Schematic integration of forward genomic selection in a northern conifer breeding program. The example is matched to an average, existing white spruce improvement program. Population size and exact number of years needed for different steps may vary from species to species and particular program needs (compare with Li and Dungey 2018). In the case of a newly starting program, phenotyping and GS model building could be made ~5 years earlier compared with the material used in the present study. Selection intensity for deployed stock could be further enhanced if seedlots from previous crossings are still available for genotyping and forward selection based on genomic predictions. GS genomic selection, SE somatic embryogenesis.

Figure 3 provides an example of how forward genomic prediction with polycross mating may integrate into an early breeding cycle for a typical northern conifer. We suggest polycross mating after GS model building in an existing progeny test and a first forward selection step. Resulting seeds would be brought to germination and DNA would be extracted from cotyledons and candidates genotyped. Using genomic profiles, GS models would be used to rank the seedlings according to their predicted breeding values and to identify superior selections for clonal propagation by means of somatic emblings or rooted cuttings (Lelu-Walter et al. 2013; Park et al. 2016). This step would implicate a second step of forward GS to further increase selection intensity or facilitate multi-trait selection (Lenz et al. 2020). In parallel, selections are planted in clonal archives for breeding and for (top-) grafting of seed orchard material. We estimate that first plants obtained with somatic embryogenesis would be available for reforestation 7 years after the first forward GS step; plants originating from rooted cuttings would be available a couple of years later. Hence, the combination of breeding and selection populations, as in forward selection, is particularly interesting for the application of GS when selection is followed by mass propagating of highly superior material.

Operationally, the use of pollen mixtures demands less effort and the reduced risk of male-female incompatibility in a polymix crossing design will shorten the time needed for completing the breeding cycle by 2 years compared with full-sib crosses. But despite the application of genomic prediction, the entire breeding cycle in white spruce will request nearly 15 years (Fig. 3), given that the species reaches sexual maturity and produces flowers only after 12–15 years. This delay could be made shorter with the use of appropriate gibberellin flowering induction treatments (Pharis et al. 1986).

Besides the ease and cost-effective implementation of polycross mating design compared with partial diallel mating design, the large number of realized mating for both females and males allows for a more reliable estimation of additive effects and breeding values, and provides good opportunities for genetic gain (Lambeth et al. 2001). Polycross mating has since been advocated as a cost-effective approach for forward selection (e.g. Doerksen and Herbinger 2010; Vidal et al. 2017). The main disadvantage of the polycross was the lack of pedigree tracing and inbreeding control that can nowadays be overcome by using genetic markers for pedigree reconstruction (Bouffier et al. 2019) or by using the genomic relationship matrix in GS models. With polycross mating, more parents can be crossed with equal breeding efforts, as compared with the partial diallel mating design. In addition, there are more chances of best-parent by best-parent mating and more choices of high value crosses from which selections can be made to obtain the desired genetic diversity level (Lambeth et al. 2001). A potential drawback of the polycross is the strongly unequal representation of pollen donors as seen in the present (Fig. 2a) and previous studies (Doerksen and Herbinger 2008; Vidal et al. 2015). As such, the precision of genetic parameters estimates and of predicted genetic gain would be higher if all males contributed equally. Nevertheless, standard errors of heritability estimates were similar to those of the full-sib progeny test (Table 2), and precision would further improve with larger sample sizes. The unequal representation of males also means that not all possible crosses were represented in the offspring generation (443 out of 722 possible biparental crosses in our sample) and that some families contained fewer individuals, giving less possibilities for forward selections, genetic gains, and inbreeding management. But it would be operationally unrealistic to perform 722 controlled full-sib crosses, and even more in a large, operational breeding program. Therefore, in the era of GS, the polycross mating design appears to be very well suited for early breeding cycles, keeping a relatively large genetic base as breeding programs are advancing, and offering the possibility to focus on additive effects and possibly non-additive effects (Gamal El-Dien et al. 2016) by testing many crosses while tracing full pedigree relationships. Later breeding cycles may thereafter rely completely or in part on controlled full-sib crosses to breed most promising individuals with higher specific combining ability and to capture non-additive effects in selections.

## Conclusions

In the present study, we evaluated GS modelling in a polycross mating design and compared the findings with results obtained from an independent full-sib (partial diallel) mating design based on the same parents. In the polycross, the recovery of full pedigree indicated a very variable number of offspring for each pollen donor which, when neglected, led to overestimated genetic parameters and inexact predictions of genetic merit. GS models using a few thousand SNPs representative of as many gene loci distributed across the 12 spruce chromosomes led to improved prediction accuracies of genetic merit of offspring and thereby, offered a more precise approach compared with simple pedigree reconstruction. We did not find any advantage to use operationally more demanding and cost-intensive full-sib crosses. The accuracy of genomic predictions in the full-sib versus the polycross progeny test was significantly higher for wood density, but was in a similar range for all other traits. However, the polycross mating design allowed for higher genetic gains per unit time while maintaining genetic diversity and incurring reduced breeding costs.

Besides optimizing genetic gain as the economic motivation, breeders need to manage coancestry in each selection and deployment decision. Genomic prediction in association with polycross mating design appears especially suitable for early breeding cycles of outcrossed conifers where this approach allows for rather high selection intensities while testing a large number of realized matings. The full potential of GS was observed in forward selection schemes while no particular advantage to increase gain or accuracy of predictions was found for backward selection. Decreasing genotyping costs make it affordable to genotype accurately hundreds or thousands of individuals and include GS into advanced tree breeding programs, allowing to hasten breeding cycles and increase selection intensity (Beaulieu et al. 2014; Park et al. 2016; Lenz et al. 2017). This is especially true for spruces, considering the continued development and improvement of clonal reproduction techniques such as somatic embryogenesis, and their application at the operational scale (Park et al. 2016). The positive results obtained herein with testing GS for hastening and improving selection efficiency in the context of polycross mating designs is a further indication of the usefulness of GS approaches in tree breeding.

## Supplementary information

Supplementary tables

Supplementary figures

Appendix 1 - Genotyping array

Appendix 2 (Dominance models)

## Data Availability

In order to comply with Intellectual Property Policies (IPP) of participating governmental institutions in this work, the supporting phenotyping data is not deposited into a public domain. The original data is stored in the institutions’ databases and may be shared upon request to the corresponding author according to our IPP. The SNP genotyping chip is provided in Appendix 1. The SNP genotyping dataset is currently subject to another publication and will be deposited into the public domain afterwards. Until then, it may be shared upon request to the corresponding author.
