# Publisher Correction: Genomic prediction for hastening and improving efficiency of forward selection in conifer polycross mating designs: an example from white spruce

**DOI:** 10.1038/s41437-022-00501-9

**Published:** 2022-02-16

**Authors:** Patrick R. N. Lenz, Simon Nadeau, Aïda Azaiez, Sébastien Gérardi, Marie Deslauriers, Martin Perron, Nathalie Isabel, Jean Beaulieu, Jean Bousquet

**Affiliations:** 1 Natural Resources Canada, Canadian Wood Fibre Centre, 1055 rue Du PEPS, P.O. Box 10380, Québec, QC G1V 4C7 Canada; 2 Canada Research Chair in Forest Genomics, Institute of Systems and Integrative Biology, and Centre for Forest Research, Université Laval, 1030 Avenue de la Médecine, Québec, QC G1V 0A6 Canada; 3 Ministère des Forêts, de la Faune et des Parcs, Gouvernement du Québec, Direction de la recherche forestière, 2700 rue Einstein, Québec, QC G1P 3W8 Canada; 4 Natural Resources Canada, Laurentian Forestry Centre, 1055 rue Du PEPS, P.O. Box 10380, Québec, QC G1V 4C7 Canada

**Keywords:** Plant breeding, Quantitative trait

Correction to: *Heredity* 10.1038/s41437-019-0290-3, published online 22 January 2020

Due to a processing error, the column headers of table 3 are displaced.

The original article has been corrected.

