# Supplementary material for: Genomic prediction for hastening and improving efficiency of forward selection in conifer polycross mating designs: an example from white spruce
Source: Heredity (Edinb). 2020 Jan 22;124(4):562–78. doi: 10.1038/s41437-019-0290-3 (PMC7080810; doi:10.1038/s41437-019-0290-3)
Supplement: Supplementary file 1 — Supplementary tables [file 41437_2019_290_MOESM1_ESM.docx]

**Table S1.** **Overview of experimental design**: number of sites, crosses, parents and trees in the polycross and the full-sib progeny test.

|  | Polycross progeny test | Full-sib progeny test |
| --- | --- | --- |
| Nb. of sites | 3: NOR, VAL, WAT | 2: ASS, SCA |
| Exp. design: Randomized complete blocks | interlocked,  non-continuous four tree plots | five-tree row plots |
| Nb. of crosses | 38 | 54 |
| Nb. of parents involved in crosses | 54^1^ | 42 |
| Nb. of offspring trees available | 892 | 1513 |
| Nb. of offspring trees in analyses (equation [3]) | 856^2^ | Ten random samples of 856 trees^3^ |
| Status number (*Ns*) in the offspring generation | 38.46 | 36.19 |
| Age after plantation at trait assessment | 19: growth, acoustic velocity 18: average density | 16: all traits |

^1^Genotypes of 46 polycross parents were used in analyses as eight pollen donors could not be recovered.

^2^Trees remaining after discarding 23 offspring due to pollen contamination, 11 offspring not assigned to the expected mother and two trees with phenotypic outlier values.

^3^Trees were randomly selected to obtain a comparable number of offspring as in the polycross test.

**Table S2.** **Polycross progeny test (*n* = 856 trees):** variance components^1^ and their significance^2^ obtained from models including only additive effects^3^. The models tested are: “ABLUP partial pedigree” is the conventional pedigree-based model using the $\boldsymbol{A}$ matrix computed from the partial pedigree with known mothers, but unknown fathers; “ABLUP full pedigree” is the model using the $\boldsymbol{A}$matrix computed from the full polycross pedigree with known mothers and retrieved fathers; “GBLUP” is the genomic selection model using the realized additive genomic relationship matrix ($\boldsymbol{G}$).

|  | Height | DBH | Volume | Acoustic velocity | Wood density |
| --- | --- | --- | --- | --- | --- |
| **ABLUP partial pedigree** |  |  |  |  |  |
| $\hat{\sigma}_{a}^{2}$ | 5065.9 (2021.9) *** | 143.9 (58.3) *** | 203.0 (79.1) *** | 0.06 (0.02) *** | 312.7 (104.4) *** |
| $\hat{\sigma}_{sa}^{2}$ | 1964.3 (1692.7) | 33.3 (49.0) | 76.4 (63.2) | 0.00 (0.01) | 24.2 (65.4) |
| $\hat{\sigma}_{e}^{2}$ | 9727.6 (2004.8) | 347.6 (59.6) | 349.1 (76.7) | 0.06 (0.02) | 403.6 (96.6) |
|  |  |  |  |  |  |
| **ABLUP full pedigree** |  |  |  |  |  |
| $\hat{\sigma}_{a}^{2}$ | 3557.9 (1315.8) *** | 125.4 (42.9) *** | 160.0 (53.7) *** | 0.05 (0.01) *** | 326.1 (83.5) *** |
| $\hat{\sigma}_{sa}^{2}$ | 2009.0 (1073.3) * | 44.6 (31.7) | 62.2 (38.8) * | 0.00 (0.01) | 0.00 (0.00) |
| $\hat{\sigma}_{e}^{2}$ | 11447.9 (1156.2) | 365.0 (37.0) | 420.3 (44.6) | 0.06 (0.01) | 441.3 (53.3) |
|  |  |  |  |  |  |
| **GBLUP** |  |  |  |  |  |
| $\hat{\sigma}_{a}^{2}$ | 3419.9 (1140.6) *** | 109.8 (33.4) *** | 140.6 (42.0) *** | 0.05 (0.01) *** | 279.6 (55.9) *** |
| $\hat{\sigma}_{sa}^{2}$ | 2318.0 (1070.3) ** | 47.5 (30.9) | 74.7 (39.0) * | 0.00 (0.00) | 0.0 (0.0) |
| $\hat{\sigma}_{e}^{2}$ | 11239.5 (1009.9) | 371.9 (32.0) | 420.8 (37.8) | 0.07 (0.01) | 469.7 (37.4) |

^1^$\hat{\sigma}_{a}^{2}$ = additive variance; $\hat{\sigma}_{sa}^{2}$ = site-by-additive interaction variance; $\hat{\sigma}_{e}^{2}$ = residual variance.

^2^Significance tests for variance components: * = *P* < 0.05; ** = *P* < 0.01; *** = *P* < 0.001.

^3^The model fitted is described in equation [3] in the manuscript.

**Table S3.** **Polycross progeny test (*n* = 856 trees):** variance components^1^ and their significance^2^, heritability, and type-B genetic correlation estimates obtained from models including additive and dominance effects^3^. The models tested are: “ABLUP full pedigree” is the model using the $\boldsymbol{A}$matrix computed from the full polycross pedigree with known mothers and retrieved fathers; “GBLUP” is the genomic selection model using the realized additive genomic relationship matrix ($\boldsymbol{G}$).

|  | Height | DBH | Volume | Acoustic velocity | Wood density |
| --- | --- | --- | --- | --- | --- |
| **ABLUP full pedigree** | | | | | |
| $\hat{\sigma}_{a}^{2}$ | 3423.4 (1320.1) *** | 122.4 (43.2) *** | 153.2 (54.2) *** | 0.05 (0.01) *** | 316.1 (83.2) *** |
| $\hat{\sigma}_{sa}^{2}$ | 1966.7 (1064.0) * | 40.7 (32.2) | 60.5 (39.4) | 0.00 (0.01) | 0.00 (0.00) |
| $\hat{\sigma}_{d}^{2}$ | 2185.2 (2768.3) | 46.2 (99.5) | 127.5 (121.6) | 0.00 (0.00) | 32.9 (135.6) |
| $\hat{\sigma}_{sd}^{2}$ | 0.0 (0.0) | 92.1 (164.9) | 45.3 (196.3) | 0.00 (0.00) | 318.1 (221.7) |
| $\hat{\sigma}_{e}^{2}$ | 9419.0 (2791.7) | 233.3 (139.6) | 255.8 (167.0) | 0.06 (0.01) | 98.8 (194.4) |
| $\hat{h}_{ind}^{2}$ | 0.20 (0.07) | 0.23 (0.07) | 0.24 (0.08) | 0.44 (0.09) | 0.41 (0.09) |
| $\hat{d}_{ind}^{2}$ | 0.13 (0.16) | 0.09 (0.19) | 0.20 (0.19) | 0.00 (0.00) | 0.04 (0.18) |
| $\hat{H}_{ind}^{2}$ | 0.33 (0.17) | 0.32 (0.19) | 0.44 (0.19) | 0.44 (0.09) | 0.46 (0.19) |
| $\hat{r}_{B ADD}$ | 0.64 (0.17) | 0.75 (0.18) | 0.72 (0.16) | 0.91 (0.10) | 1.00 (0.00) |
| $\hat{r}_{B GENO}$ | 0.74 (0.15) | 0.56 (0.39) | 0.73 (0.41) | 0.91 (0.10) | 0.52 (0.24) |
|  |  |  |  |  |  |
| **GBLUP** |  |  |  |  |  |
| $\hat{\sigma}_{a}^{2}$ | 3396.8 (1140.0) *** | 110.8 (33.8) *** | 143.5 (42.6) *** | 0.05 (0.01) *** | 279.3 (55.9) *** |
| $\hat{\sigma}_{sa}^{2}$ | 2300.0 (1064.3) ** | 49.7 (31.1) | 76.0 (38.7) * | 0.00 (0.00) | 0.0 (0.0) |
| $\hat{\sigma}_{d}^{2}$ | 1044.5 (1162.4) | 61.0 (37.9) | 74.5 (44.2) | 0.00 (0.00) | 0.0 (0.0) |
| $\hat{\sigma}_{sd}^{2}$ | 0.0 (0.0) | 0.0 (0.0) | 0.0 (0.0) | 0.01 (0.01) | 110.8 (80.4) |
| $\hat{\sigma}_{e}^{2}$ | 10253.3 (1441.4) | 310.4 (45.8) | 345.7 (53.7) | 0.06 (0.01) | 361.4 (82.8) |
| $\hat{h}_{ind}^{2}$ | 0.20 (0.06) | 0.21 (0.06) | 0.22 (0.06) | 0.41 (0.06) | 0.37 (0.06) |
| $\hat{d}_{ind}^{2}$ | 0.06 (0.07) | 0.11 (0.07) | 0.12 (0.07) | 0.00 (0.00) | 0.00 (0.00) |
| $\hat{H}_{ind}^{2}$ | 0.26 (0.09) | 0.32 (0.09) | 0.34 (0.09) | 0.41 (0.06) | 0.37 (0.06) |
| $\hat{r}_{B ADD}$ | 0.60 (0.16) | 0.69 (0.16) | 0.65 (0.15) | 0.93 (0.09) | 1.00 (0.00) |
| $\hat{r}_{B GENO}$ | 0.66 (0.15) | 0.78 (0.13) | 0.74 (0.12) | 0.83 (0.19) | 0.72 (0.15) |

^1^$\hat{\sigma}_{a}^{2}$ = additive variance; $\hat{\sigma}_{sa}^{2}$ = site-by-additive interaction variance; $\hat{\sigma}_{d}^{2}$ = dominance variance; $\hat{\sigma}_{sd}^{2}$ = site-by-dominance interaction variance; $\hat{\sigma}_{e}^{2}$ = residual variance; $\hat{h}_{ind}^{2}$ = individual narrow-sense heritability; $\hat{d}_{ind}^{2}$ = proportion of phenotypic variance due to dominance; $\hat{H}_{ind}^{2}$ = individual broad-sense heritability (additive + dominance effects); $\hat{r}_{B ADD}$ = type-B additive genetic correlation; $\hat{r}_{B GENO}$ = type-B total genetic correlation (additive + dominance effects).

^2^Significance tests for variance components: * = *P* < 0.05; ** = *P* < 0.01; *** = *P* < 0.001.

^3^The model fitted is described in equation [7] in Appendix 2.

**Table S4.** **Full-sib progeny test:** variance components^1^ and their significance^2^, heritability, and type-B genetic correlation estimates obtained from the genomic selection models (GBLUP) including only additive effects^3^. Results are presented for the full dataset (1513 trees) and for 10 random samples of 856 trees out of 1513.

|  | Height | DBH | Volume | Acoustic velocity | Wood density |
| --- | --- | --- | --- | --- | --- |
| **GBLUP: *n* = 1513 trees** | | | | | |
| $\hat{\sigma}_{a}^{2}$ | 1363.9 (406.0) *** | 21.2 (12.5) | 21.2 (8.5) ** | 0.04 (0.01) *** | 263.6 (43.9) *** |
| $\hat{\sigma}_{sa}^{2}$ | 758.0 (330.2) *** | 27.6 (12.5) *** | 16.5 (7.4) *** | 0.00 (0.00) | 31.2 (19.5) * |
| $\hat{\sigma}_{e}^{2}$ | 5642.0 (301.6) | 264.2 (12.5) | 141.9 (7.2) | 0.08 (0.00) | 453.0 (25.7) |
| $\hat{h}_{ind}^{2}$ | 0.18 (0.05) | 0.07 (0.04) | 0.12 (0.05) | 0.33 (0.05) | 0.35 (0.05) |
| $\hat{r}_{B}$ | 0.64 (0.14) | 0.43 (0.22) | 0.56 (0.18) | 0.93 (0.06) | 0.89 (0.06) |
|  |  |  |  |  |  |
| **GBLUP: 10 random samples of *n* = 856 trees out of 1513** | | | | | |
| $\hat{\sigma}_{a}^{2}$ | 1436.69 (556.59) * | 18.94 (16.59) | 19.26 (11.04) | 0.04 (0.01) *** | 267.75 (61.13) *** |
| $\hat{\sigma}_{sa}^{2}$ | 701.18 (443.94) * | 28.32 (17.86) * | 16.24 (10.16) * | 0.01 (0) | 43.06 (32.17) |
| $\hat{\sigma}_{e}^{2}$ | 5713.6 (438.66) | 265.86 (17.51) | 143.51 (10.1) | 0.08 (0.01) | 440.44 (38.12) |
| $\hat{h}_{ind}^{2}$ | 0.18 (0.07) | 0.06 (0.05) | 0.11 (0.06) | 0.35 (0.07) | 0.36 (0.07) |
| $\hat{r}_{B}$ | 0.67 (0.19) | 0.39 (0.33) | 0.53 (0.26) | 0.89 (0.09) | 0.86 (0.1) |

^1^$\hat{\sigma}_{a}^{2}$ = additive variance; $\hat{\sigma}_{sa}^{2}$ = site-by-additive interaction variance; $\hat{\sigma}_{e}^{2}$ = residual variance; $\hat{h}_{ind}^{2}$ = individual narrow-sense heritability; $\hat{r}_{B}$ = type-B genetic correlation.

^2^Significance tests for variance components: * = *P* < 0.05; ** = *P* < 0.01; *** = *P* < 0.001.

^3^The model fitted is described in equation [3] in the manuscript.

**Table S5.** **Full-sib progeny test:** variance components^1^ and their significance^2^, heritability, and type-B genetic correlation estimates obtained from the genomic selection models (GBLUP) including additive and dominance effects^3^. Results are presented for the full dataset (1513 trees) and for 10 random samples of 856 trees out of 1513.

|  | Height | DBH | Volume | Acoustic velocity | Wood density |
| --- | --- | --- | --- | --- | --- |
| **GBLUP: *n* = 1513 trees** | | | | | |
| $\hat{\sigma}_{a}^{2}$ | 1120.2 (415.0) ** | 14.0 (12.8) | 16.5 (8.7) * | 0.04 (0.01) *** | 245.2 (44.4) *** |
| $\hat{\sigma}_{sa}^{2}$ | 786.2 (337.0) ** | 23.8 (13.7) * | 15.8 (8.1) ** | 0.00 (0.00) | 29.4 (21.0) |
| $\hat{\sigma}_{d}^{2}$ | 655.2 (304.5) | 18.6 (16.1) | 11.9 (8.6) | 0.01 (0.00) | 50.0 (32.6) |
| $\hat{\sigma}_{sd}^{2}$ | 0.00 (0.00) | 18.7 (20.8) | 4.8 (10.7) | 0.00 (0.00) | 9.7 (35.3) |
| $\hat{\sigma}_{e}^{2}$ | 5177.2 (349.1) | 236.7 (15.6) | 129.8 (8.8) | 0.07 (0.01) | 411.1 (32.8) |
| $\hat{h}_{ind}^{2}$ | 0.14 (0.05) | 0.04 (0.04) | 0.09 (0.05) | 0.31 (0.05) | 0.33 (0.05) |
| $\hat{d}_{ind}^{2}$ | 0.08 (0.04) | 0.06 (0.05) | 0.07 (0.05) | 0.07 (0.04) | 0.07 (0.04) |
| $\hat{H}_{ind}^{2}$ | 0.23 (0.05) | 0.10 (0.05) | 0.16 (0.06) | 0.37 (0.05) | 0.40 (0.06) |
| $\hat{r}_{B ADD}$ | 0.59 (0.16) | 0.37 (0.31) | 0.51 (0.22) | 0.92 (0.06) | 0.89 (0.07) |
| $\hat{r}_{B GENO}$ | 0.69 (0.12) | 0.43 (0.22) | 0.58 (0.19) | 0.93 (0.05) | 0.88 (0.10) |
|  |  |  |  |  |  |
| **GBLUP: 10 random samples of 856 trees out of 1513** | | | | | |
| $\hat{\sigma}_{a}^{2}$ | 1283.8 (577.0) * | 14.3 (17.4) | 15.4 (11.0) | 0.04 (0.01) *** | 252.1 (62.5) *** |
| $\hat{\sigma}_{sa}^{2}$ | 669.3 (480.6) | 22.5 (19.6) | 14.1 (10.4) | 0.00 (0.01) | 35.3 (33.9) |
| $\hat{\sigma}_{d}^{2}$ | 497.9 (629.9) | 14.9 (26.0) | 8.7 (13.1) | 0.01 (0.01) | 38.1 (54.3) |
| $\hat{\sigma}_{sd}^{2}$ | 235.0 (864.9) | 27.5 (34.4) | 8.6 (18.3) | 0.00 (0.01) | 47.0 (71.5) |
| $\hat{\sigma}_{e}^{2}$ | 4358.0 (646.6) | 246.0 (29.1) | 98.1 (13.4) | 0.08 (0.01) | 398.0 (59.3) |
| $\hat{h}_{ind}^{2}$ | 0.18 (0.08) | 0.04 (0.05) | 0.11 (0.07) | 0.30 (0.07) | 0.33 (0.07) |
| $\hat{d}_{ind}^{2}$ | 0.07 (0.08) | 0.05 (0.06) | 0.06 (0.08) | 0.06 (0.07) | 0.05 (0.07) |
| $\hat{H}_{ind}^{2}$ | 0.25 (0.09) | 0.09 (0.07) | 0.17 (0.09) | 0.37 (0.08) | 0.38 (0.08) |
| $\hat{r}_{B ADD}$ | 0.66 (0.23) | 0.38 (0.48) | 0.51 (0.32) | 0.90 (0.12) | 0.87 (0.12) |
| $\hat{r}_{B GENO}$ | 0.67 (0.24) | 0.38 (0.30) | 0.52 (0.27) | 0.86 (0.16) | 0.79 (0.13) |

^1^$\hat{\sigma}_{a}^{2}$ = additive variance; $\hat{\sigma}_{sa}^{2}$ = site-by-additive interaction variance; $\hat{\sigma}_{d}^{2}$ = dominance variance; $\hat{\sigma}_{sd}^{2}$ = site-by-dominance interaction variance; $\hat{\sigma}_{e}^{2}$ = residual variance; $\hat{h}_{ind}^{2}$ = individual narrow-sense heritability; $\hat{d}_{ind}^{2}$ = proportion of phenotypic variance due to dominance; $\hat{H}_{ind}^{2}$ = individual broad-sense heritability (additive + dominance effects); $\hat{r}_{B ADD}$ = type-B additive genetic correlation; $\hat{r}_{B GENO}$ = type-B total genetic correlation (additive + dominance effects).

^2^Significance tests for variance components: * = *P* < 0.05; ** = *P* < 0.01; *** = *P* < 0.001.

^3^The model fitted is described in equation [7] in Appendix 2.
