# Supplementary material for: Genomic prediction for hastening and improving efficiency of forward selection in conifer polycross mating designs: an example from white spruce
Source: Heredity (Edinb). 2020 Jan 22;124(4):562–78. doi: 10.1038/s41437-019-0290-3 (PMC7080810; doi:10.1038/s41437-019-0290-3)
Supplement: Supplementary file 2 — Supplementary figures [file 41437_2019_290_MOESM2_ESM.docx]

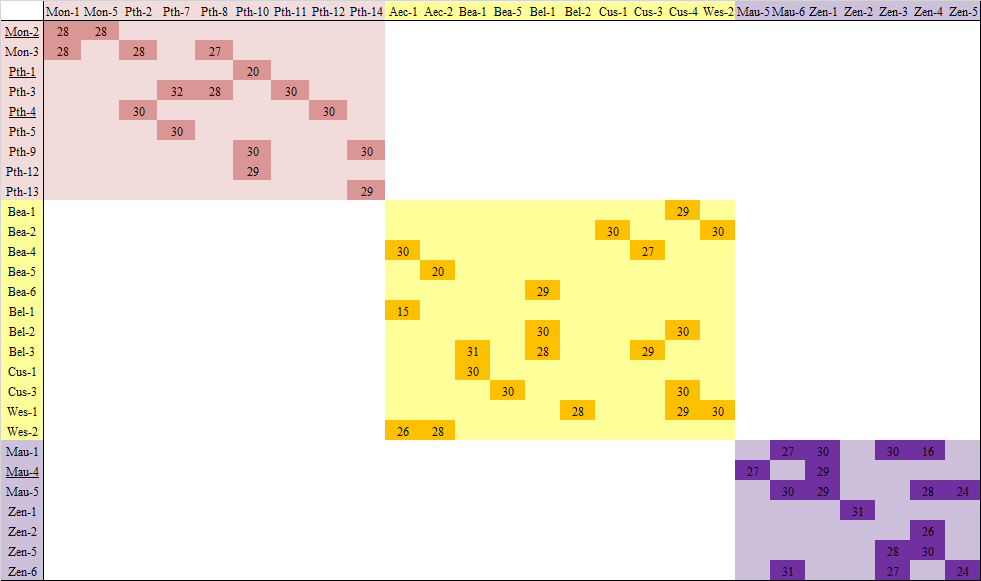


**Figure S1. Full-sib progeny test:** partial diallel mating design. Mothers and fathers are listed in rows and columns respectively. The four parents that were not part of the polycross progeny test are underlined. The number in cells indicate the number of progeny genotyped for each cross used in data analyses (total of 1513 trees).


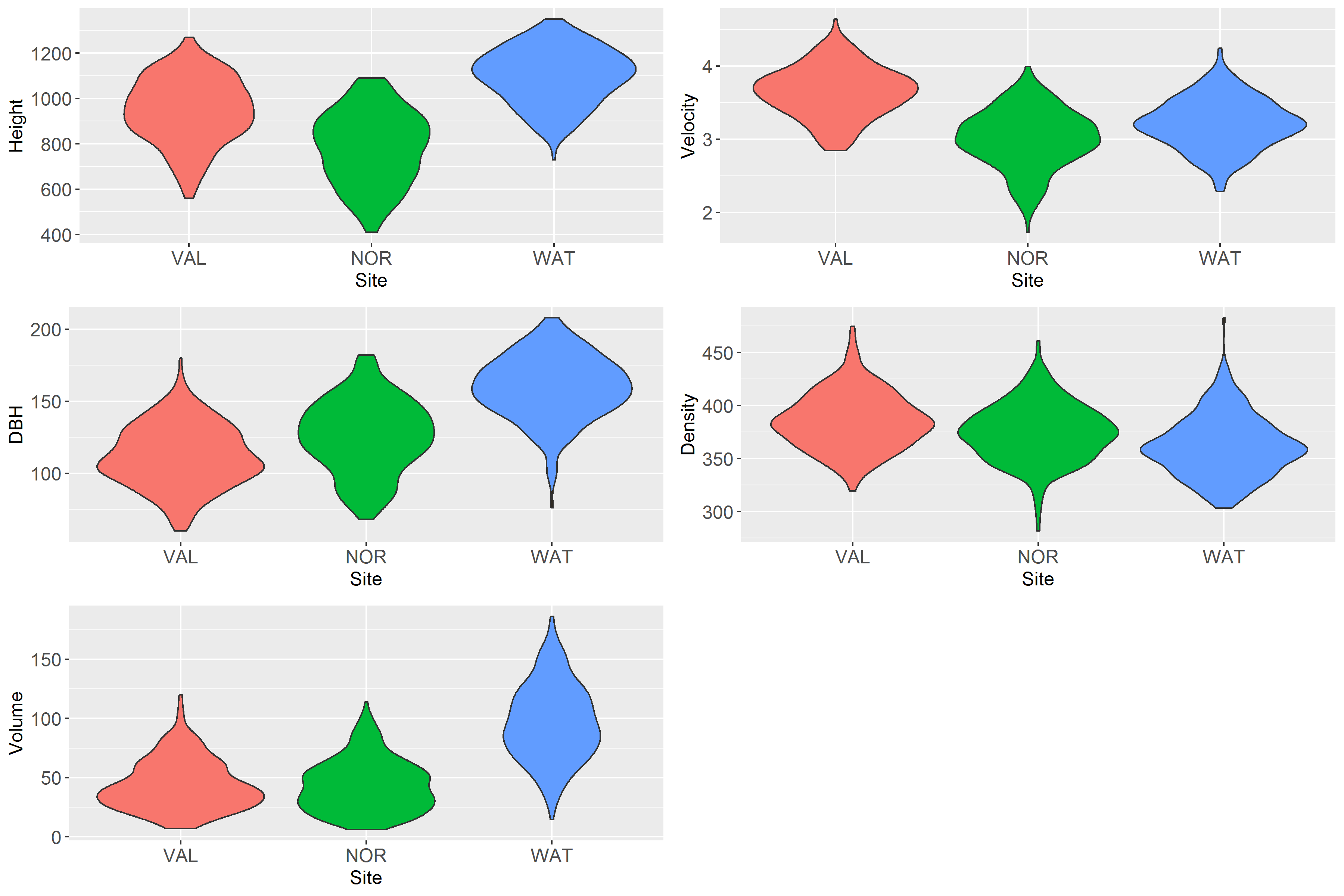


**Figure S2.** **Polycross progeny test:** violin plots grouped by sites for the traits assessed in this study. See Table 1 in the manuscript for full description of traits.


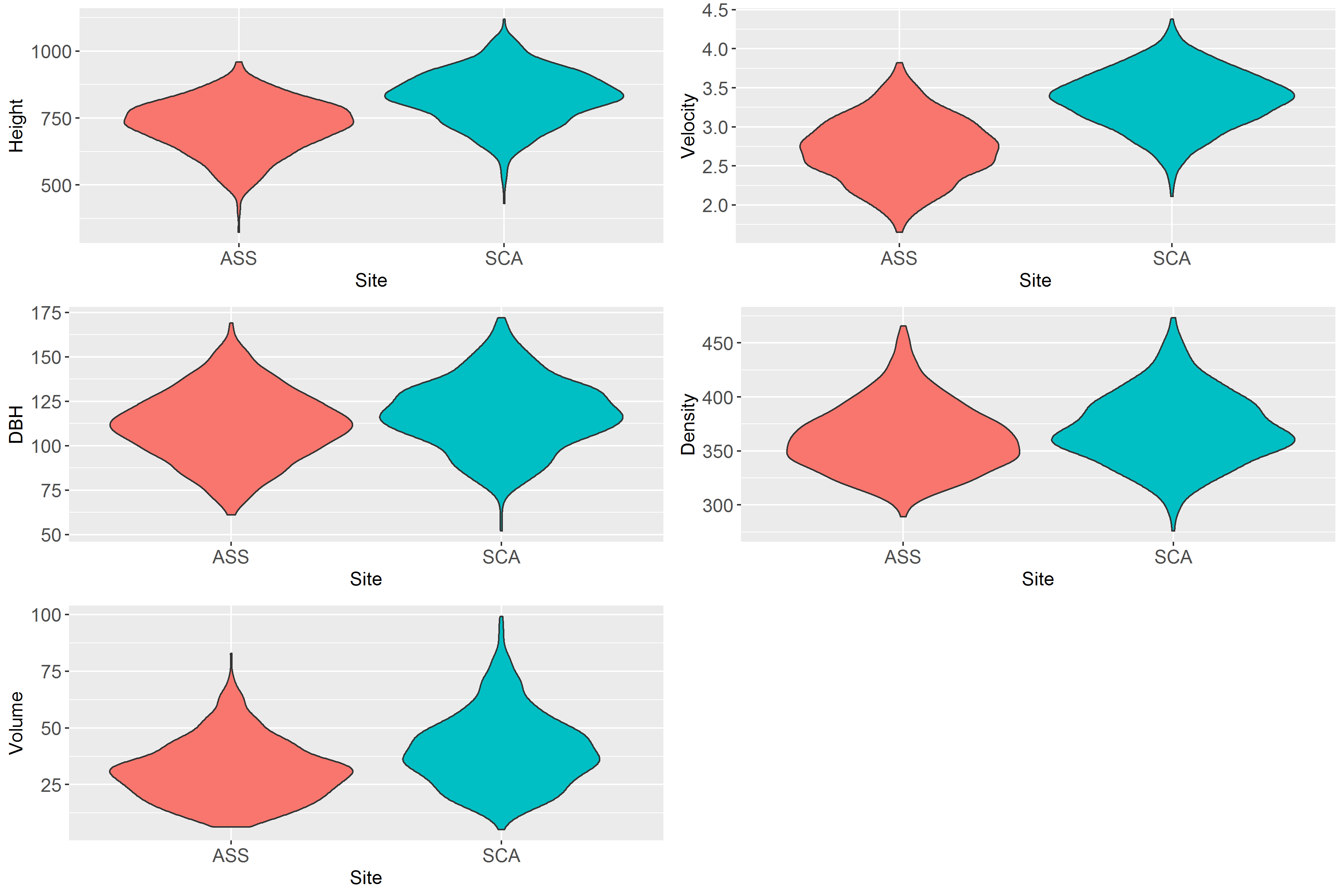


**Figure S3.** **Full-sib progeny test:** violin plots grouped by sites for the traits assessed in this study. See Table 1 in the manuscript for full description of traits.


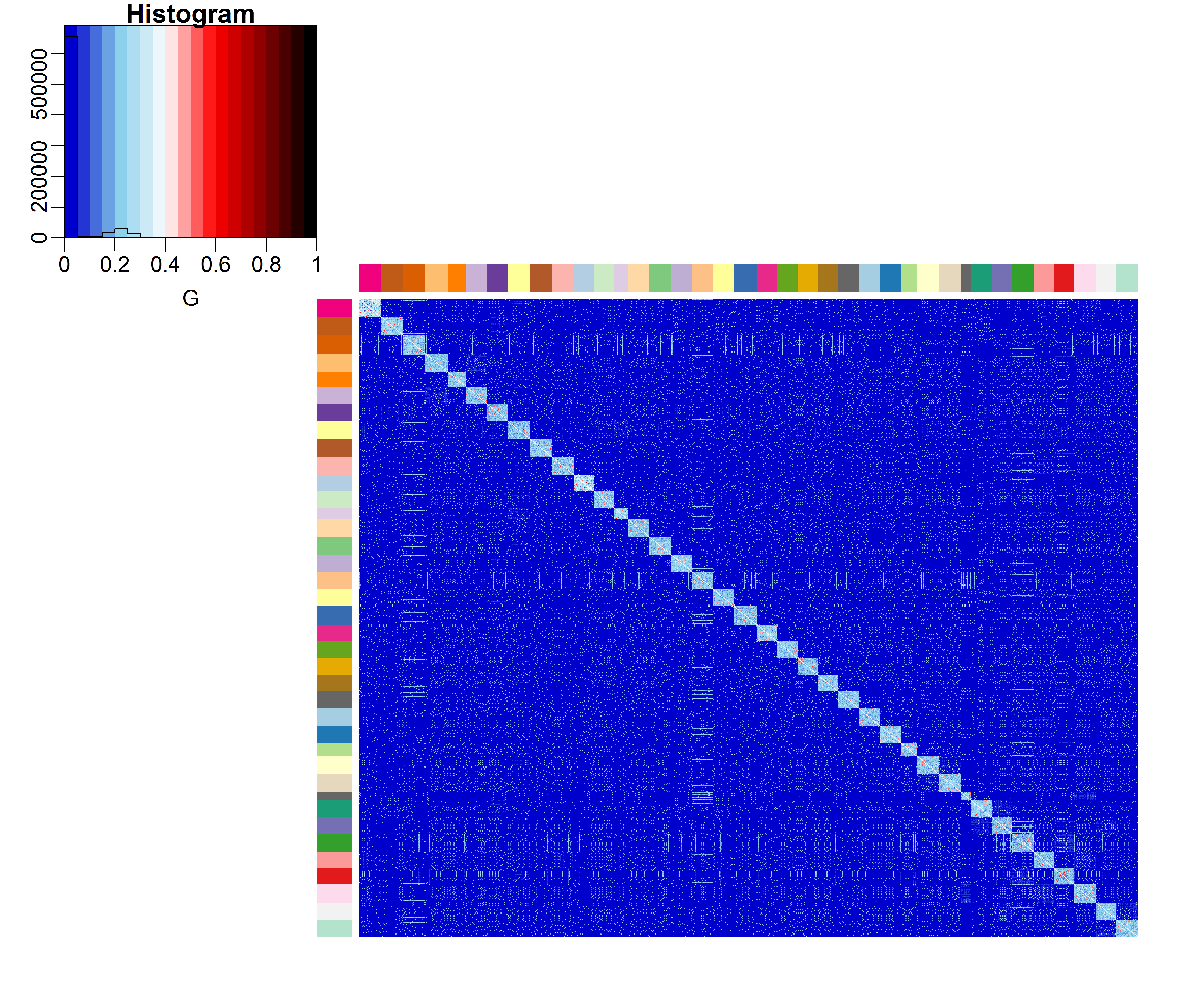


**Figure S4. Polycross progeny test:** realized genomic relationship matrix ($\boldsymbol{G}$) for the 856 trees. The histogram on the top left represents the colour key for the values of G. The side colours on the x and y-axes represent the 38 maternal polycross families.

| (A) | 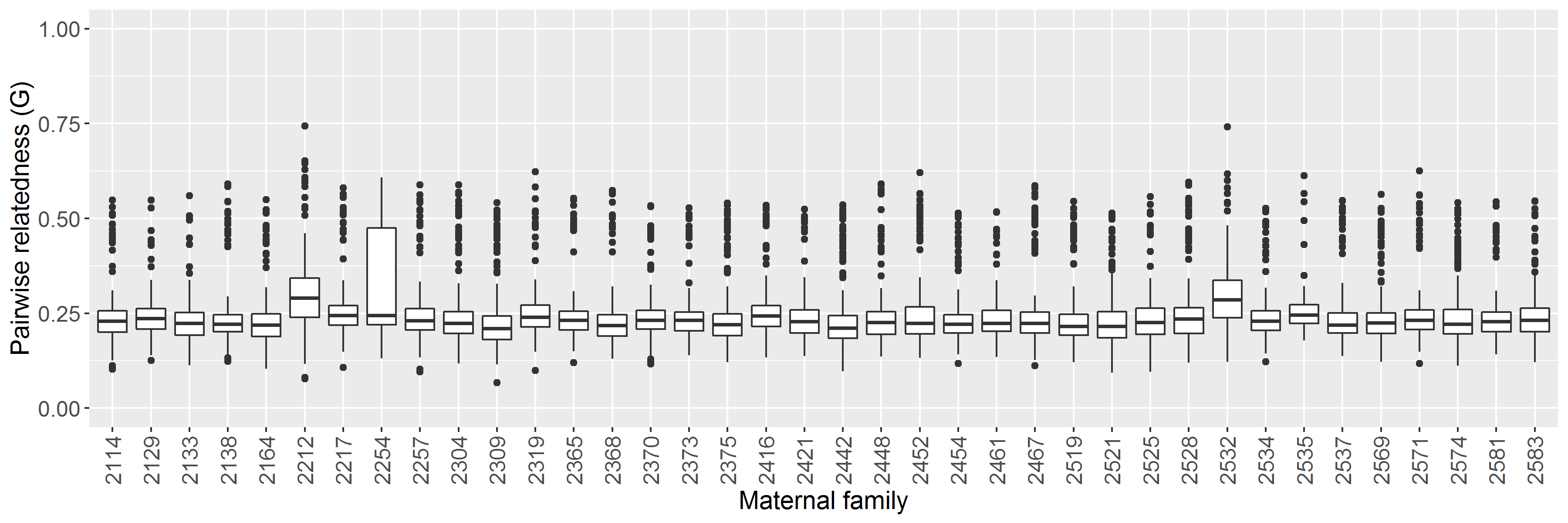 |
| --- | --- |
| (B) | 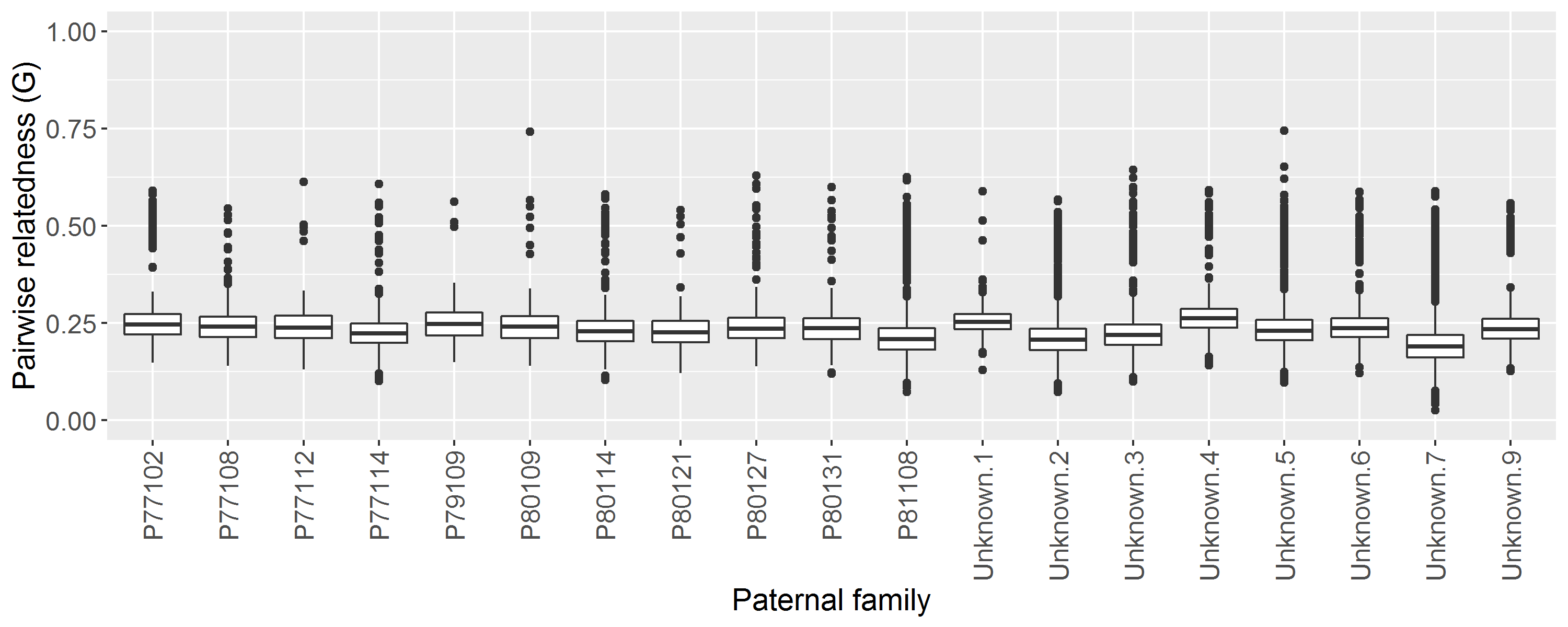 |

**Figure S5. Polycross progeny test:** pairwise realized genomic relationship ($\boldsymbol{G}$) between trees within each of (A) the 38 maternal families and (B) the 19 recovered paternal families from paternity assignment analyses.


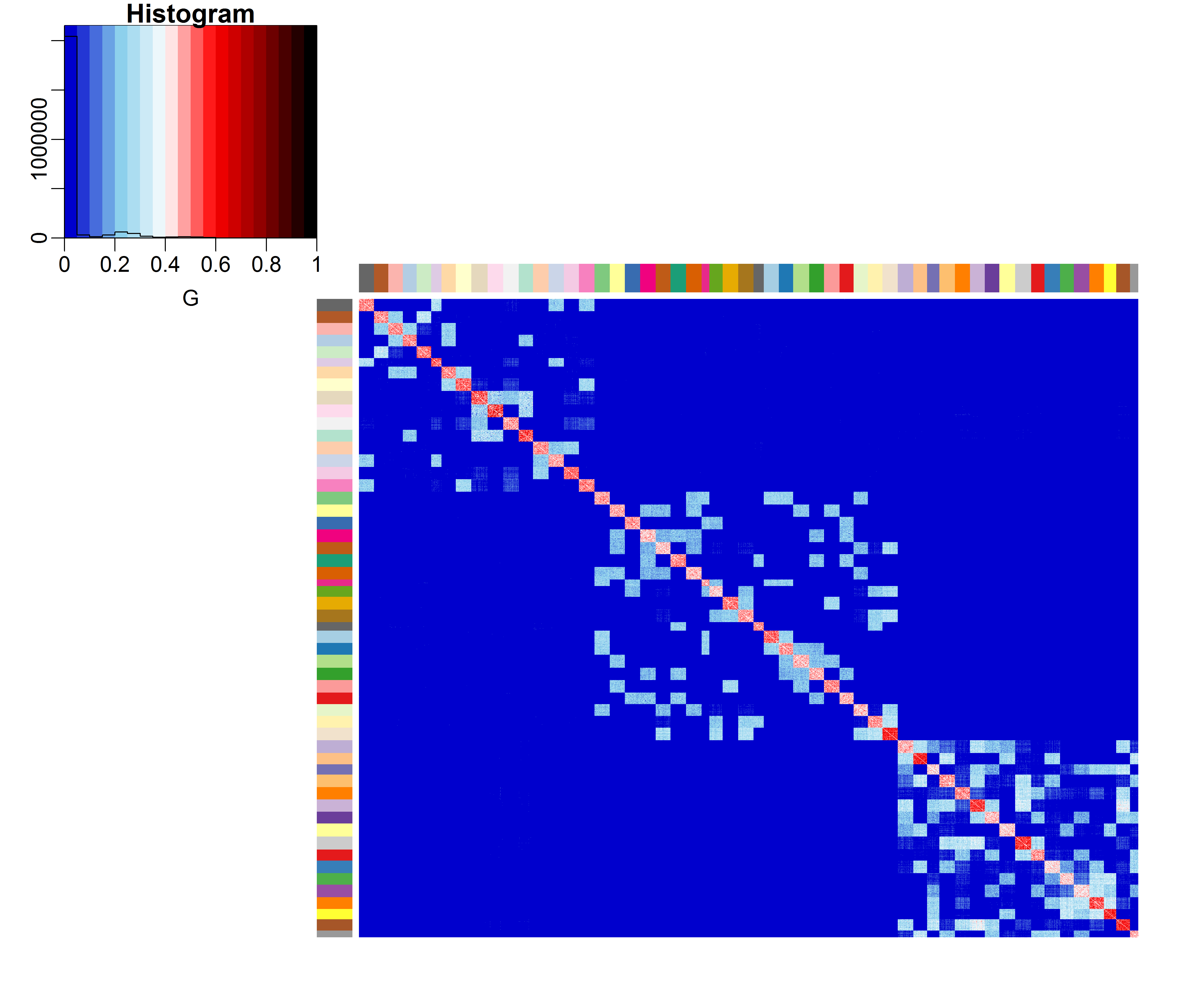


**Figure S6. Full-sib progeny test:** realized genomic relationship matrix ($\boldsymbol{G}$) for the 1513 trees. The histogram on the top left represents the colour key for the values of G. The side colours on the x and y-axes represent the 54 full-sib families.


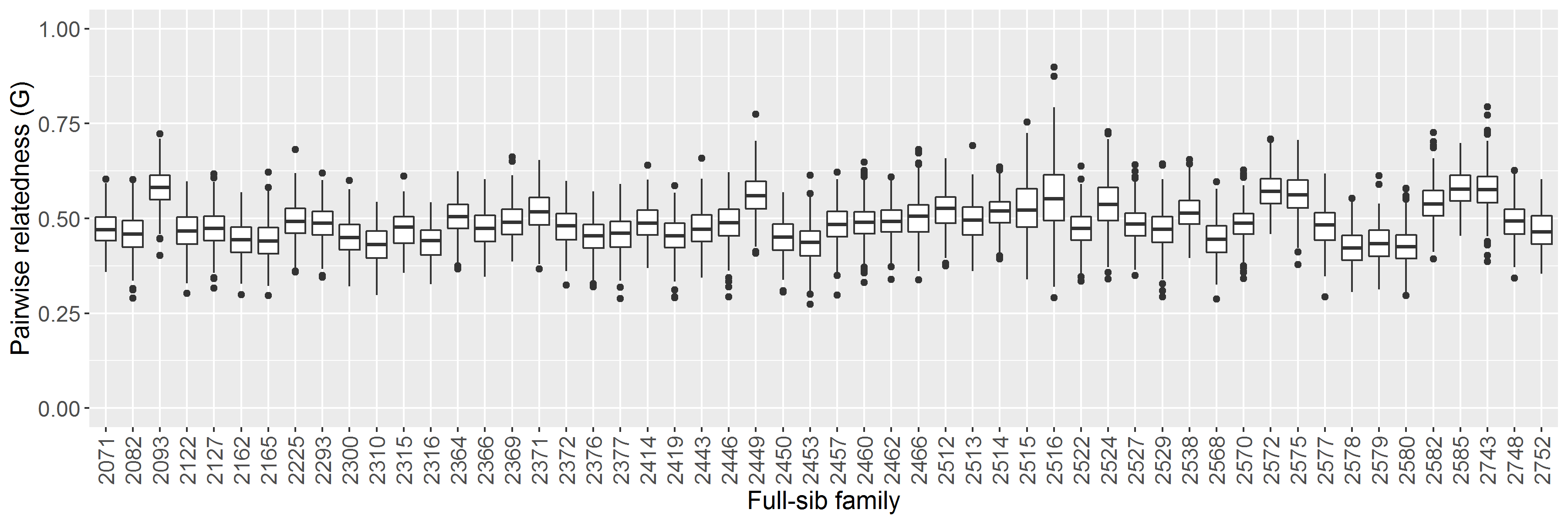


**Figure S7. Full-sib progeny test:** pairwise realized genomic relationship ($\boldsymbol{G}$) between trees within each of the 54 full-sib families.


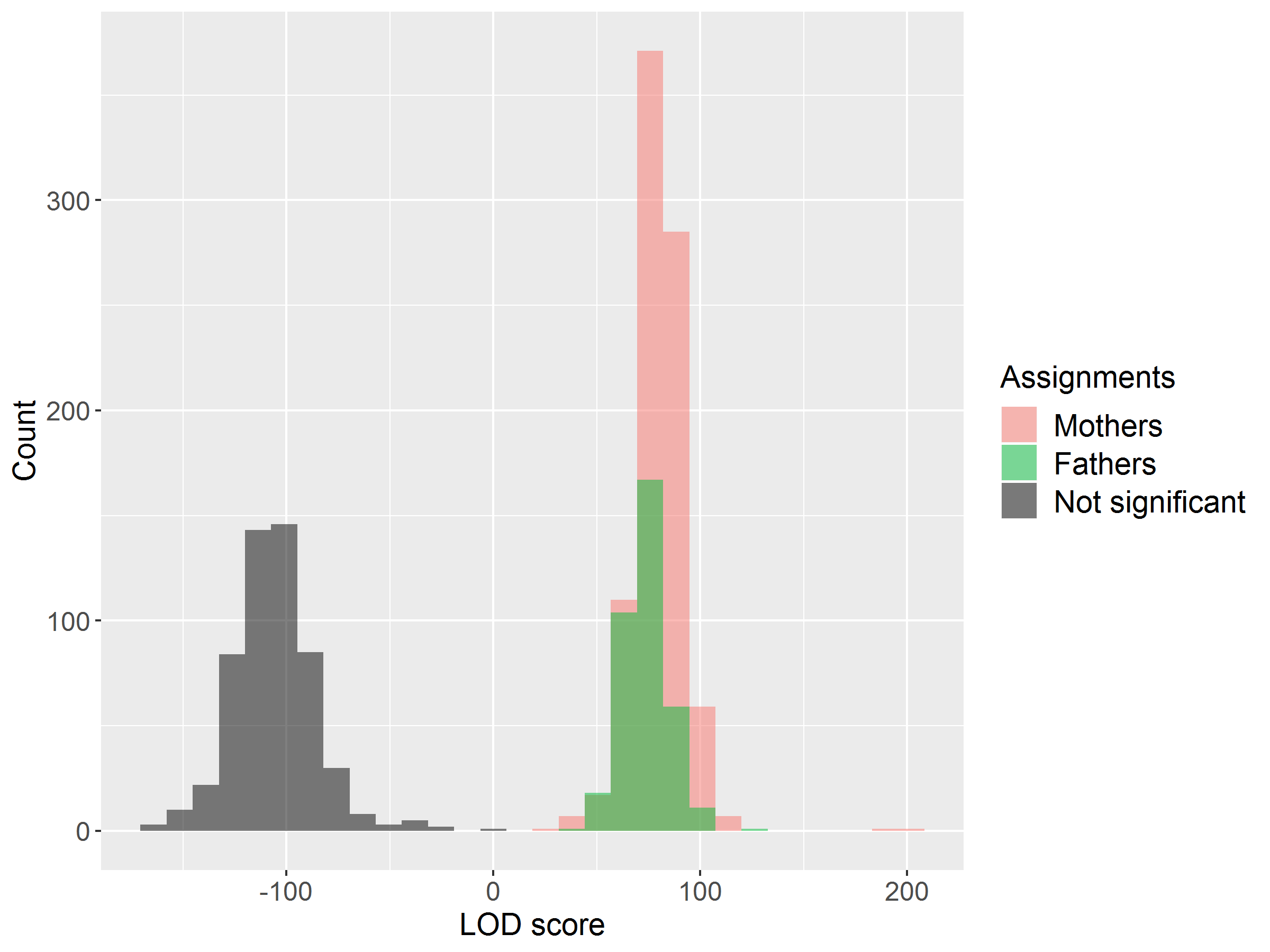


**Figure S8.** **Polycross progeny test:** overlapping histograms of LOD scores for the most likely candidate mother and father for each offspring determined by CERVUS. LOD scores of successfully assigned mothers are shown in pink (*n* = 881); sucessfully assigned fathers in green (*n* = 361); and fathers that could not be assigned in grey (*n* = 520).


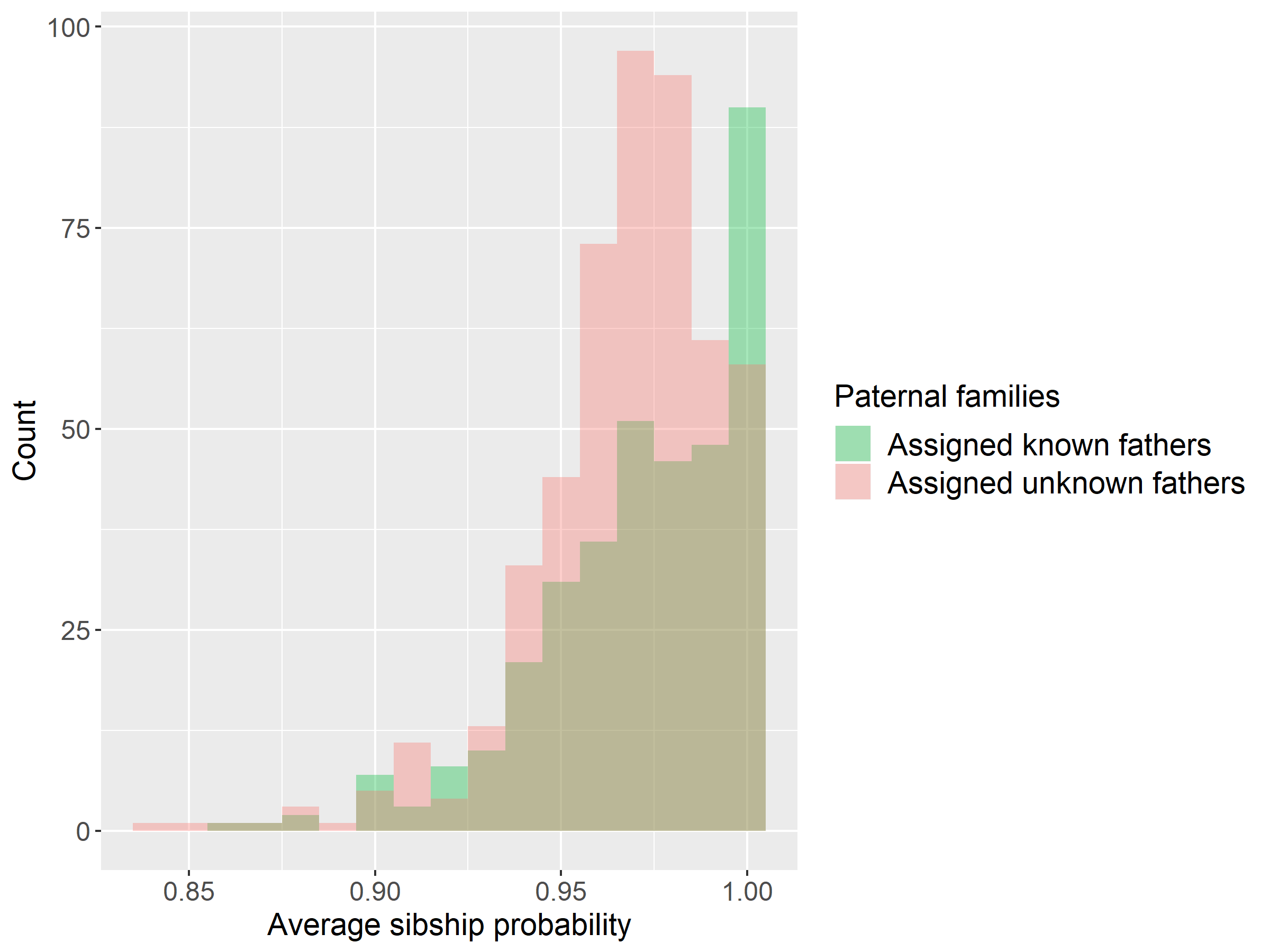


**Figure S9.** **Polycross progeny test:** overlapping histograms of average sibship probability within paternal families. Offspring assigned to known fathers (the 11 genotyped fathers) are shown in green and offspring assigned to unknown fathers (the 8 fathers that were not genotyped) are shown in pink. The average sibship probability for each offspring within a paternal family was computed from all pairwise sibship probabilities between an offspring and all his siblings as inferred from the software COLONY.


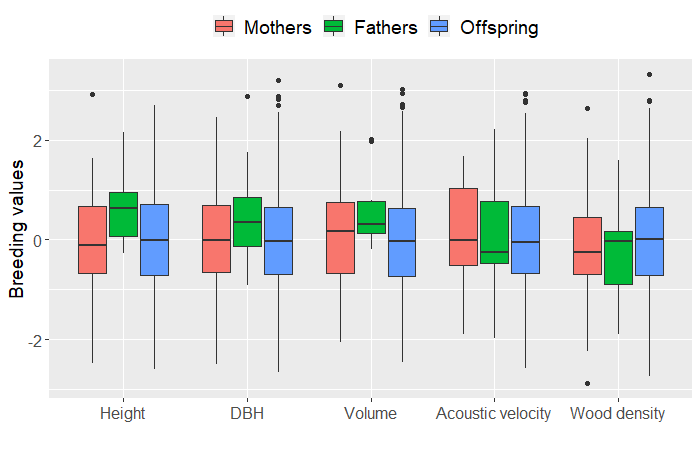


**Figure S10. Polycross progeny test:** estimated breeding values using GBLUP of the 38 mothers, the 11 genotyped father, and the 856 offspring.
