# Supplementary material for: Genomic prediction for hastening and improving efficiency of forward selection in conifer polycross mating designs: an example from white spruce
Source: Heredity (Edinb). 2020 Jan 22;124(4):562–78. doi: 10.1038/s41437-019-0290-3 (PMC7080810; doi:10.1038/s41437-019-0290-3)
Supplement: Supplementary file 4 — Appendix 2 (Dominance models) [file 41437_2019_290_MOESM4_ESM.docx]

**Appendix 2**

The following individual-tree mixed GBLUP models including additive and dominance genetic effects were fitted in ASReml-R v.3.0:

[7] $\boldsymbol{y}=\mu+\boldsymbol{X\beta}+\boldsymbol{Z}_{\mathbf{1}}\boldsymbol{a}+\boldsymbol{Z}_{\mathbf{2}}\boldsymbol{sa}+\boldsymbol{Z}_{\mathbf{3}}\boldsymbol{d}+\boldsymbol{Z}_{\mathbf{4}}\boldsymbol{sd}+\boldsymbol{e}$,

where $\boldsymbol{y}$ is the phenotype; $\boldsymbol{\beta}$, $\boldsymbol{a}$, $\boldsymbol{sa}$, and $\boldsymbol{e}$ are as described in equation [3] in the manuscript; $\boldsymbol{d}$ is the random dominance genetic effect, with $\boldsymbol{d}\sim N\left( 0,\sigma_{d}^{2}\boldsymbol{A}_{\boldsymbol{Dom}} \right)$ for the ABLUP model and $\boldsymbol{d}\sim N\left( 0,\sigma_{d}^{2}\boldsymbol{G}_{\boldsymbol{Dom}} \right)$ for the GBLUP model; $\boldsymbol{sd}$ is the random interaction of site with dominance genetic effects, with $\boldsymbol{sd}\sim N(0,\sigma_{sd}^{2}\boldsymbol{I}_{\boldsymbol{s}}\boldsymbol{A}_{\boldsymbol{Dom}}$) for the ABLUP model and $\boldsymbol{sd}\sim N(0,\sigma_{sd}^{2}\boldsymbol{I}_{\boldsymbol{s}}\boldsymbol{G}_{\boldsymbol{Dom}}$) for the GBLUP model. $\boldsymbol{A}_{\boldsymbol{Dom}}$ is the dominance relationship matrix obtained from the pedigree using function makeD of the R package nadiv. $\boldsymbol{G}_{\boldsymbol{Dom}}$ is the realized dominance relationship matrix calculated following Vitezica et al (2013). Individual narrow-sense heritability was calculated as:

[8] $\hat{h}_{ind}^{2}={\hat{\sigma}_{a}^{2}}/{(\hat{\sigma}_{a}^{2}+\hat{\sigma}_{sa}^{2}+\hat{\sigma}_{d}^{2}+ \hat{\sigma}_{sd}^{2}+\hat{\sigma}_{e}^{2})}$

The proportion of phenotypic variance due to dominance was calculated as:

[9] $\hat{d}_{ind}^{2}={\hat{\sigma}_{d}^{2}}/{(\hat{\sigma}_{a}^{2}+\hat{\sigma}_{sa}^{2}+\hat{\sigma}_{d}^{2}+ \hat{\sigma}_{sd}^{2}+\hat{\sigma}_{e}^{2})}$

The individual broad-sense heritability was calculated as:

[10] $\hat{H}_{ind}^{2}={{(\hat{\sigma}}_{a}^{2}+\hat{\sigma}_{d}^{2})}/{(\hat{\sigma}_{a}^{2}+\hat{\sigma}_{sa}^{2}+\hat{\sigma}_{d}^{2}+ \hat{\sigma}_{sd}^{2}+\hat{\sigma}_{e}^{2})}$

The type-B additive genetic correlation was calculated as:

[11] $\hat{r}_{B ADD}={\hat{\sigma}_{a}^{2}}/{(\hat{\sigma}_{a}^{2}+\hat{\sigma}_{sa}^{2})}$

The type-B total genetic correlation was calculated as:

[12] $\hat{r}_{B GENO}={{(\hat{\sigma}_{a}^{2}+\hat{\sigma}}_{d}^{2})}/{({\hat{\sigma}_{a}^{2}+\hat{\sigma}_{sa}^{2}+\hat{\sigma}}_{d}^{2}+\hat{\sigma}_{sd}^{2})}$
